# CSF d18:1 sphingolipid species in Parkinson disease and dementia with Lewy bodies with and without *GBA1* variants

**DOI:** 10.1038/s41531-024-00820-0

**Published:** 2024-10-24

**Authors:** Stefanie Lerche, Isabel Wurster, Enza Maria Valente, Micol Avenali, Daniela Samaniego, Marta Martínez-Vicente, Jorge Hernández-Vara, Ariadna Laguna, Andrea Sturchio, Per Svenningsson, Nicholas P. France, Carrolee Barlow, Sethu Sankaranarayanan, Kathrin Brockmann

**Affiliations:** 1 Center of Neurology, Department of Neurodegeneration and Hertie-Institute for Clinical Brain Research, University of Tuebingen, Tuebingen, Germany; 2 German Center for Neurodegenerative Diseases, University of Tuebingen, Tuebingen, Germany; 3 Edmond J. Safra Fellow in Movement Disorders, New York, NY USA; 4 Department of Molecular Medicine, University of Pavia, Pavia, Italy; 5 IRCCS Mondino Foundation, Pavia, Italy; 6 Department of Brain and Behavioural Sciences, University of Pavia, Pavia, Italy; 7 Neurodegenerative Diseases Research Group, Vall d’Hebron Research Institute (VHIR)-Network center for Biomedical Research in Neurodegenerative Diseases (CIBERNED), Barcelona, Spain; 8 Aligning Science Across Parkinson’s (ASAP) Collaborative Research Network, Chevy Chase, MD USA; 9 Department of Clinical Neuroscience, Karolinska Institute, Stockholm, Sweden; 10 ESCAPE Bio, South San Francisco, CA USA

**Keywords:** Parkinson's disease, Neurodegeneration

## Abstract

Variants in *GBA1* result in dysregulated sphingolipids. We investigated five CSF d18:1 sphingolipid species in a longitudinal multicenter cohort comprising people with Parkinson’s Disease and Dementia with Lewy bodies with and without *GBA1* variants and healthy controls. We found no increase of sphingolipid species in heterozygous *GBA1* variant participants and no effect on development of cognitive impairment. Thus, CSF d18:1 sphingolipids are not suitable as state markers in Parkinson’s Disease.

## Introduction

Bi-allelic variants in the glucocerebrosidase gene (*GBA1*) cause Gaucher Disease (GD). Heterozygous variants in *GBA1* represent the most important genetic risk factor for alpha-synucleinopathies with Lewy-body pathology, Parkinson’s Disease (PD), and Dementia with Lewy Bodies (DLB)^1^. *GBA1* encodes the lysosomal enzyme glucocerebrosidase (GCase), which metabolizes glucosylceramides (GlcCer) and glucosylsphingosines (GlcSph). Experimental evidence suggests that *GBA1* variants result in lower GCase activity and cause a build-up of GlcCer^2^ which also impairs lysosomal function and the degradation of alpha-synuclein (α-synuclein)^3^. In addition to these directly linked metabolites, downstream products of the broader sphingolipid pathway (including sphingosine (Sph) and sphingosine-1-phosphate (S1P)) seem also affected due to *GBA1* variants^4^. Data from *α-synuclein/GBA1* transgenic mice and cell cultures support this notion indicating that not only GlcCer but also GlcSph, Sph, and S1P promote the aggregation of α-synuclein^5–7^. Notably, GlcSph has been shown to cause α-synuclein oligomerization and aggregation even more prominently than GlcCer^6^. The link between *GBA1* with PD and DLB offers various therapeutic targets which, in turn, require reliable (fluid)biomarkers to monitor trait, state, target engagement, and/or response to therapy. In GD, GlcCer, although it is the direct substrate and more abundant than GlcSph, is not a useful biomarker^8^. In contrast, plasma levels of GlcSph show good correlation with disease severity and are used to monitor response to therapy. A recent study reported elevated plasma levels of GlcSph d18:1 isoform in participants with and without PD carrying a heterozygous *GBA1* N370S variant compared to PD and healthy participants without pathogenic *GBA1* or *LRRK2* G2019S variants^9^. All other assessed sphingolipids (total GlcCer, Cer, Galactosylceramide (GalCer), and Galactosylsphingosine (GalSph)) showed similar mean plasma levels between groups. While this finding is interesting, several key questions remain unanswered: 1. Is this peripheral GlcSph signature representative of the central nervous system and similarly found in CSF of heterozygous *GBA1* variant carriers? 2. The N370S variant is one of several important *GBA1* variants. How do sphingolipid profiles look in other *GBA1* variant groups? 3. Apart from PD, heterozygous *GBA1* variants are prominently associated with DLB. Are sphingolipid profiles found in PD similar or even more prominent in DLB participants carrying *GBA1* variants as both entities represent a biological continuum? To address these questions, we investigated 5 CSF d18:1 sphingolipid species (Cer (d18:1/18:0), GlcCer (d18:1/18:0), SphM (d18:1/18:0), GlcSph (d18:1) and GalSph (d18:1)) in a multicenter cohort with 152 PD and 37 DLB participants with and without heterozygous *GBA1* variants. We also included 5 samples from PD participants who were bi-allelic carriers of *GBA1* variants as “positive controls” and 40 healthy elderly as control participants. We estimated the effect of sphingolipid CSF levels on longitudinal development of cognitive impairment in PD.

## CSF levels of d18:1 sphingolipids by GBA1 genotype and GBA1 severity

We combined PD and DLB groups and stratified by *GBA1* genotype. Both, PD_GBA_ + DLB_GBA_ as well as PD_GBA_WT_ + DLB_GBA_WT_ groups had lower levels of GalSph compared to HC. All other assessed sphingolipid species did not differ between *GBA1* and WT status; Table 1.

**Table 1:** CSF levels of sphingolipids stratified by *GBA1* genotype

	Healthy control *n* = 40	PD_GBA_WT_ + DLB_GBA_WT_ *n* = 105	PD_GBA_ + DLB_GBA_ *n* = 84	PD_Gaucher_ *n* = 5	*p*-value
Ceramide [pmol/ml]	3.83 ± 1.21	3.76 ± 1.41	4.01 ± 1.44	4.34 ± 1.60	0.547^a^
Glucosylceramide [pmol/ml]	0.85 ± 0.21	0.90 ± 0.27	0.92 ± 0.25	1.03 ± 0.17	0.526^a^
Glucosylsphingosine [pmol/ml]	0.014 ± 0.003	0.013 ± 0.004	0.014 ± 0.004	0.052 ± 0.044^***,###,§§§^	<0.001^a^
Galactosylsphingosine [pmol/ml]	0.170 ± 0.057	0.151 ± 0.045^*^	0.151 ± 0.036^*^	0.160 ± 0.042	0.108^a^
Sphingomyelin [pmol/ml]	328 ± 73	333 ± 100	342 ± 90	377 ± 92	0.647^a^

Data are shown as mean ± standard deviation.

^a^ANCOVA with age as co-variable; ^*^ versus HC, ^#^ versus PD_GBA_WT_ + DLB_GBA_WT_; ^§^ versus PD_GBA_ + DLB_GBA_

*p < 0.05; **p < 0.01; ***p ≤ 0.001

Subanalysis of PD and DLB stratified by *GBA1* variant severity (GBA_WT_, GBA_risk_, GBA_mild_, GBA_severe_) revealed no significant differences in levels of sphingolipid species in pmol/ml (Cer: 3.76 vs. 4.18 vs. 3.86 vs. 3.65; GlcCer: 0.90 vs. 0.94 vs. 0.92 vs. 0.86; GlcSph: 0.013 vs. 0.014 vs. 0.015 vs. 0.014; GalSph: 0.15 vs. 0.16 vs. 0.15 vs. 0.13; SphM: 333 vs. 346 vs. 346 vs. 324; *p* > 0.05 respectively); Supplemental Fig. 1.

## CSF levels of d18:1 sphingolipids by GBA1 genotype and disease group

DLB_GBA_WT_ had lower levels of Cer than PD_GBA_, PD_GBA_WT_, and HC (*p* < 0.05, respectively). DLB_GBA_ and DLB_GBA_WT_ had lower levels of GalSph than HC (*p* < 0.05 and *p* < 0.01). Overall, but purely numerically, DLB_GBA_ had higher levels of all sphingolipid species compared to DLB_GBA_WT_.

In PD, levels of GlcSph were numerically higher in PD_GBA_ compared to PD_GBA_WT_ while levels of all other sphingolipid species showed no clear trend.

**Fig. 1:**
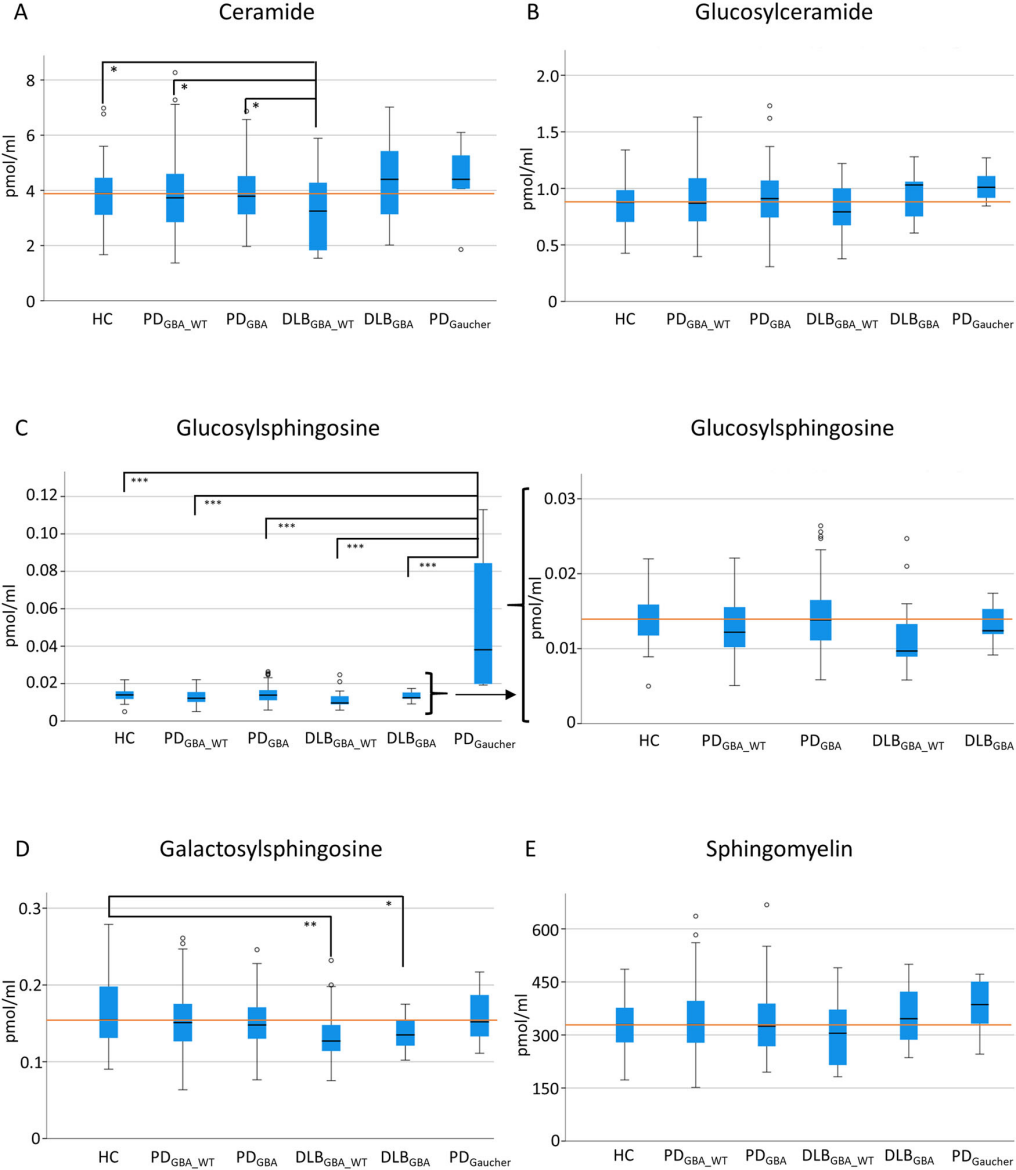
CSF levels of sphingolipids stratified by *GBA1* genotype and disease group. DLB_GBA_WT_ patients had lower levels of ceramide than HC, PD_GBA_WT_, and PD_GBA_ patients (**A**). PD_Gaucher_ patients had higher levels of glucosylsphingosine than HC, PD_GBA_WT_, PD_GBA_, DLB_GBA_WT_, and DLB_GBA_ (**C**). DLB_GBA_WT_ and DLB_GBA_ patients had lower levels of galactosylsphingosine than HC (**D**). There is no difference in levels of glucosylceramide and sphingomyelin between the patient groups (**B** + **E**). The red line indicates median levels of healthy controls, respectively. *p < 0.05; **p < 0.01;***p < 0.001.

As proof-of-concept, PD_Gaucher_ by far showed the highest levels of GlcSph (4-fold increase) compared to all other groups; Fig. 1.

## Correlation between CSF d18:1 sphingolipids and clinical measurements

In PD_GBA_WT_ + DLB_GBA_WT_, higher levels of GalSph (*ρ* = −0.276, *p* = 0.005) were associated with lower H&Y. There were no significant correlations between sphingolipid species and clinical scores (H&Y, UPDRS-III and MoCA) in the combined group of *GBA1* variant carriers PD_GBA_ + DLB_GBA_. There were no correlations between levels of sphingolipid species and *GBA1* severity.

## Longitudinal association of baseline CSF d18:1 sphingolipid tertiles with cognitive impairment

Kaplan-Meier analyses revealed no differences in reaching the milestone cognitive impairment when comparing the lowest with the highest tertile of any sphingolipid species, neither in the combined group of all PD patients nor when analysing tertiles of PD_GBA_ and PD_GBA_WT_ separately; Supplemental Fig. 2.

By measuring targeted CSF d18:1 isoforms of various sphingolipid species (d18:1 GlcSph, GalSph, d18:1, 18:0 Cer, GlcCer, and SphM) in a multi-center cohort comprising 152 PD and 37 DLB participants with and without various heterozygous *GBA1* variants and 40 HC we found no clear increase of the key substrates GlcCer and GlcSph when comparing *GBA1* variant carriers with their WT comparator group. Notably, levels in PD and DLB with and without *GBA1* variant were within the range of healthy elderly. Although PD_GBA_ and DLB_GBA_ had numerically higher mean levels of GlcSph compared to PD_GBA_WT_ and DLB_GBA_WT_, these small differences seem not adequate for stratification of *GBA1* variant carriers to enter clinical trials. Thereby, our present CSF data do not robustly support findings from the recent study in plasma which reported significantly elevated levels of d18:1 GlcSph in participants with and without PD carrying a heterozygous *GBA1* N370S variant compared to PD and healthy participants without *GBA1* variant^9^. It is noteworthy that we previously showed higher CSF levels of total GlcCer in PD_GBA_ compared to PD_GBA_WT_ in two different cohorts (Tuebingen *GBA1* cohort with 50% overlap with the present Tuebingen cohort, PPMI cohort)^4^. The different analytical approaches might partially explain these discrepancies. In the present study, we focused on the d18:1, 18:0 isoform using a targeted analysis with internal spiked heavy-labeled standards that enable absolute quantification with the main goal to be used as endpoints for treatment trials. Opposed to that, we used untargeted lipidomics and summed up all measured isoforms of each sphingolipid species (total Cer, total GlcCer) for the previous analysis. At that time, we identified higher CSF levels of Sph in PD_GBA_ compared to PD_GBA_WT_, but we did not measure GlcSph. Moreover, increased levels of total GlcCer in plasma have been reported in PD versus HC using a HPLC-based method, again summarizing all measured isoforms^10^. It was recently speculated that the very long-chain isoforms such as 22:0, 22:1, 23:0, 23:1, 24:0, and 24:1 are higher in *GBA1* and thereby contribute to the significance when comparing sum levels of all isoforms. While using untargeted lipidomics provides good coverage of various sphingolipid chain lengths it has lower sensitivity and only provides relative quantification. We, therefore, suggest to explore the very long-chain 22-24 isoforms in a targeted approach with absolute quantification in future studies.

We did not identify relevant correlations between sphingolipid levels and relevant clinical scores (UPDRS-III, HY, MoCA) nor did we detect differences between any sphingolipid tertile group with longitudinal development of cognitive impairment, neither in the combined group of all PD patients nor when analysing tertile groups of PD_GBA_ and PD_GBA_WT_ separately. These findings indicate that CSF levels of sphingolipids are not suited to monitor disease burden or progression in PD. This is further supported by the finding that DLB_GBA_ do not show higher sphingolipid levels than PD_GBA_. These findings are in line with the recent study in plasma where no association was found between plasma levels of any of the assessed sphingolipid species and PD disease status^9^. Thereby, and opposed to GD, (d18:1, 18:0) sphingolipid isoforms and especially GlcSph (d18:1) are not useful as state markers in PD.

The assay’s validity seems reasonable with a 4-fold increase of CSF GlcSph levels in PD_GD_. While the additional inclusion of DLB participants and the inclusion of different *GBA1* variants pose strengths of the present analyses, we acknowledge the following limitations: (I) The measurement of targeted single sphingolipid isoform (d18:1, 18:0) instead of several isoforms limits the detection of effects coming from long-chain isoforms. (II) Longitudinal measurements of sphingolipids in de-novo and enriched at-risk individuals are important to evaluate changes over time, with aging and/or with disease progression. (III) Blood-CSF pairs from the same individual at the same time point are needed. (IV) It is worth mentioning that even in the bi-allelic participants CSF levels of Cer, GlcCer, SphM, and GalSph were low in general. It would be interesting to compare these levels to those from Gaucher disease patients without PD in future studies.

## Methods

### Participants

Together, the four sites (Karolinska University Hospital, Stockholm, Sweden; IRCCS Mondino Foundation of Pavia, Italy; University of Tuebingen, Germany; Vall d’Hebron Research Institute, Barcelona, Spain) collected CSF of 79 idiopathic PD patients (PD_GBA_WT_), 73 PD patients with *GBA1* variant (PD_GBA_), 26 idiopathic DLB patients (DLB_GBA_WT_), 11 DLB patients with *GBA1* variants (DLB_GBA_) and 5 PD_Gaucher_ patients. Forty neurodegenerative healthy elderly (spouses, volunteers) served as control individuals (HC).

### Clinical investigations

Diagnosis of PD was defined according to UK Brain Bank Society Criteria or MDS clinical diagnostic criteria and diagnosis of DLB according to the DLB consortium revised consensus criteria. All participants were categorized by the modified Hoehn and Yahr Scale (H&Y), assessed with the Unified Parkinson’s Disease Rating Scale (UPDRS-III), and Montreal Cognitive Assessment (MoCA) and/or the Mini-Mental Status Examination (MMSE). Since the MoCA was available only from 2009 on, all previously obtained MMSE scores were converted into MoCA equivalent scores according to a published algorithm^11^. Presence of cognitive impairment was defined as MoCA score < 26. For demographic and clinical details see Supplemental Table 1.

### Genetic screening

Genetic screening of the Tuebingen cohort for *GBA1* variants was done by Sanger sequencing of all exons in 58% of the PD and 100% of DLB patients. In 42% of PD patients primary genetic screening was done by NeuroChip and in case of *GBA1* variants confirmation by Sanger sequencing.

Genetic screening for the Stockholm cohort was performed with pyrosequencing and TaqMan PCR with subsequent confirmation by Sanger sequencing.

Genetic screening of the majority of the Italian cohort was performed by an NGS-based method. In a minority of patients, the *GBA1* gene was tested by Sanger sequencing. Identified variants were all validated by Sanger sequencing.

Genetic screening for the Vall d’Hebron Initiative for Parkinson (VHIP) cohort was performed by an NGS-based method that combines a primary *GBA1*-specific long-range PCR with subsequent *GBA1*-exon-specific PCR and next-generation sequencing of the resulting products.

*GBA1*-subgroup classification of variant severity was based on established genotype risks reported for PD (PD_GBA_severe_, PD_GBA_mild_, PD_GBA_risk_).

An overview of *GBA1* variants is given in Supplemental Table 2.

### CSF measurement of sphingolipids

CSF Hb was measured using a sensitive ELISA assay (Bethyl Hb assay kit, Fortis Life Sciences, USA). Samples with Hb levels > 50 ng/mL were excluded in analysis.

Five CSF sphingolipid species (Cer (d18:1/18:0), GlcCer (d18:1/18:0), SphM (d18:1/18:0), GlcSph (d18:1) and GalSph (d18:1)) were measured using LC-MS/MS method at Ardena Bioanalysis BV, NL. The d18:1/18:0 variants of the specific sphingolipids were assessed since these are highly expressed in brain^12,13^. The analytical ranges were 0.500–50.0, 0.250–25.0, 25.0–2500, 0.00500–1.00, and 0.005–1.00 pmol/mL, respectively. All reference materials were obtained from Avanti Polar Lipids Inc., Alabaster AL, USA). All samples were measured blinded to clinical and genetic status.

Cer (d18:1/18:0) and spiked internal standard Cer (d18:1-d7/18:0) were isolated from 50 µL human CSF by protein precipitation with acetonitrile (ACN): isopropanol (IPA) (60:40 v/v). After precipitation, blank-, calibration-, QC-, and study samples were injected into the Chromatographic system (Shimadzu Nexera, Kyoto, Japan) on an xSelect CSH Phenyl-Hexyl column (100 × 3.0 mm (length × internal diameter), 2.5 µm (particle size), Waters, Milford, MA, USA) using isocratic elution with water: IPA:ACN:FA (formic acid) (16.5:18.5:65:0.1 (v/v/v/v)) as mobile phase. An API6500 tandem mass spectrometer (Sciex, Framingham, MA, USA) equipped with a Turbo Ion Spray probe operating in the positive multiple reaction monitoring mode was used for quantification. MS transitions were 566 → 264 and 573 → 271 (unit resolution) for Cer (d18:1/18:0) and Cer (d18:1-d7/18:0), respectively. The analytical range was 0.500–50.0 pmol/mL. Each analytical run included duplicate QC samples at three levels (QC-Low at 1.50 pmol/mL, QC-Medium at 8.00 pmol/mL, and QC-High at 40.0 pmol/mL) and a representative QC-Pool sample in quadruplicate.

GlcCer (d18:1/18:0) and SM (d18:1/18:0) and spiked internal standards GlcCer (d18:1-d5/18:1) and SM (d18:1/17:0) were isolated from 50 µL human CSF by liquid-liquid extraction (LLE) with ethyl acetate: IPA: Pentane (51:9:40 (v/v/v)). After extraction, blank-, calibration-, QC-, and study samples were injected into the Chromatographic system (Shimadzu) on a Hypersil GOLD Silica column (100 × 2.1 mm (length × internal diameter), 1.9 µm (particle size), Thermo Fisher Scientific, Waltham, MA, USA) using a gradient elution with 1 M NH4FA (ammonium formate) : FA:water: ACN (20:6:40:2000 (v/v/v/v)) as mobile phase A and 1 M NH4FA:water:ACN (5:500:10 (v/v/v)) as mobile phase B. An API6500 tandem mass spectrometer (Sciex) equipped with a Turbo Ion Spray probe operating in the positive multiple reaction monitoring mode was used for quantification. MS transitions for the analytes were 728 → 264 and 731 → 184 (unit resolution) for GlcCer (d18:1/18:0) and SM (d18:1/18:0), respectively. Transitions for the internals standards were 731 → 269 and 717 → 184, respectively. The analytical ranges were 0.250–25.0 and 25.0–2500 pmol/mL, respectively. Each analytical run included duplicate QC samples at three levels (QC-Low at 0.750/75.0 pmol/mL, QC-Medium at 4.00/400 pmol/mL, and QC-High at 20.0/2000 pmol/mL for GlcCer (d18:1/18:0) and SM (d18:1/18:0), respectively) and a representative QC-Pool sample in quadruplicate.

GlcSph (d18:1) and GalSph (d18:1) and spiked internal standards GlcSph (d18:1)-d5 and GalSph (d18:1)-d5 were isolated from 200 µL of human CSF by solid phase extraction (SPE) using 1cc 30 mg Waters Oasis MCX cartridges (Waters). In short, cartridges were equilibrated with 1 mL methanol (MeOH) and 1 mL water, after which the acidified CSF samples (0.2 mL CSF + 0.1 mL 1.0/2.0 mg/mL bovine serum albumin/ascorbic acid in phosphate buffer saline (PBS) + 0.75 mL 1% H3PO4 + 0.1 mL 2.0 mg/mL ascorbic acid in MeOH) were loaded on the cartridges. Sample were washed with 2 ×0.75 mL water and 2 × 0.75 mL MeOH after which the analytes were eluted with 2 × 0.4 mL 1% NH4FA in MeOH in a tube containing 0.1 mL of 2 mg/mL ascorbic acid in MeOH. After evaporation, extracts were redissolved in 0.0500 mL of 2 mg/mL ascorbic acid in chloroform:MeOH:UPW (5:1:0.1 (v/v/v)) after which 0.100 mL ACN was added. Blank-, calibration-, QC- and study samples were injected into the Chromatographic system (Shimadzu) on a Hypersil GOLD Silica column (100 × 2.1 mm (length × internal diameter), 1.9 µm (particle size)), Thermo Fisher Scientific) using a gradient elution with 1 M NH4FA:FA:water:ACN (20:6:40:2000 (v/v/v/v) as mobile phase A and 1 M NH4FA:water:ACN (5:500:10 (v/v/v) as mobile phase B. An API6500 tandem mass spectrometer (Sciex) equipped with a Turbo Ion Spray probe operating in the positive multiple reaction monitoring mode was used for quantification. MS transitions were 462 → 282 and 467 → 287 (unit resolution) for GlcSph (d18:1)/GalSph(d18:1) and GlcSph(d18:1)-d5/GalSph(d18:1)-d5, respectively. GlcSph(d18:1) and GalSph(d18:1) were chromatographically separated since the molecules are isomers and share similar fragments. The analytical ranges were 0.00500–1.00 pmol/mL for both analytes. Each analytical run included duplicate QC samples at three levels (QC-Low at 0.0300 pmol/mL, QC-Medium at 0.150 pmol/mL, and QC-High at 0.800 pmol/mL for both analytes) and a representative QC-Pool sample in quadruplicate.

Calibration samples were prepared fresh on the day of analysis in analyte free surrogate matrix using nine non-zero concentrations and at least one double blank (no analyte and no IS) and one blank (no IS). The calibration curves were calculated using a quadratic regression model and 1/x2 weighting. Results were accepted if the run met the predefined acceptance criteria as per FDA and EMA guidelines (1. Guidance for Industry: Bioanalytical Method Validation, U.S. Department of Health and Human Services, Food and Drug Administration, Center for Drug Evaluation and Research (CDER), Center for Veterinary Medicine (CVM), May 2018, BP. 2. Guideline on bioanalytical method validation, European Medicines Agency (EMA), Committee for Medicinal Products for Human Use (CHMP), EMEA/CHMP/EWP/192217/2009, 21 July 2011). For chromatograms of GluSph and GalSph please see supplemental material.

### Statistical analysis

Statistical analysis was performed using SPSS 26.0 (IBM). Group comparisons of continuous data were analyzed using ANOVA/ANCOVA including age as co-variate where appropriate. The five PD_Gaucher_ were included as “positive controls” for a purely descriptive comparison (not powered for robust statistical analysis).

Pearson’s correlation was used to evaluate associations between CSF levels of sphingolipids and clinical scores. As PD and DLB are a biological continuum and to explicitly asses the GBA effect we combined the two wildtype groups (PD_GBA_WT_ + DLB_GBA_WT_) and the two *GBA1* variant groups (PD_GBA_ + DLB_GBA_). As this study was exploratory, we did not correct for multiple testing. However, only correlations with at least a correlation coefficient of *ρ* > 0.20 were considered meaningful (irrespective if the *p*-value was <0.05).

For longitudinal analysis of association between CSF levels of sphingolipids and the *GBA1*-related key milestone cognitive impairment, we calculated Kaplan-Meier survival curves by stratifying all PD patients into sphingolipid tertiles based on the individual CSF level of the respective sphingolipid species. Kaplan-Meier survival curves were analyzed separately for PD_GBA_WT_ and PD_GBA_.

### Ethical approval and patient consent

The study was approved by the local Ethics Committees (Tuebingen:199/2011BO1; Pavia: PD-GEN, 16/01/2019 CE PV; Stockholm: 2016/19-31/2; Barcelona: PR(AG)170-2015, PR(AG)434-2019; ESCAPE Bio: Stitching Beoordeling Ethiek Biomedisch Onderzoek Review Board Assen, Netherlands). All participants gave written informed consent.

## Supplementary information

Supplementary material

## Data Availability

Raw data of the measurements were uploaded to Zenodo: DOI 10.5281/zenodo.13132385. Further anonymized data are available upon request to: kathrin.brockmann@uni-tuebingen.de.
