# Supplementary material for: CSF d18:1 sphingolipid species in Parkinson disease and dementia with Lewy bodies with and without GBA1 variants
Source: NPJ Parkinsons Dis. 2024 Oct 24;10:198. doi: 10.1038/s41531-024-00820-0 (PMC11502890; doi:10.1038/s41531-024-00820-0)
Supplement: Supplementary file 1 — Supplementary material [file 41531_2024_820_MOESM1_ESM.pdf]

Supplemental Figure 1: CSF levels of sphingolipids stratified by *GBA1* severity.

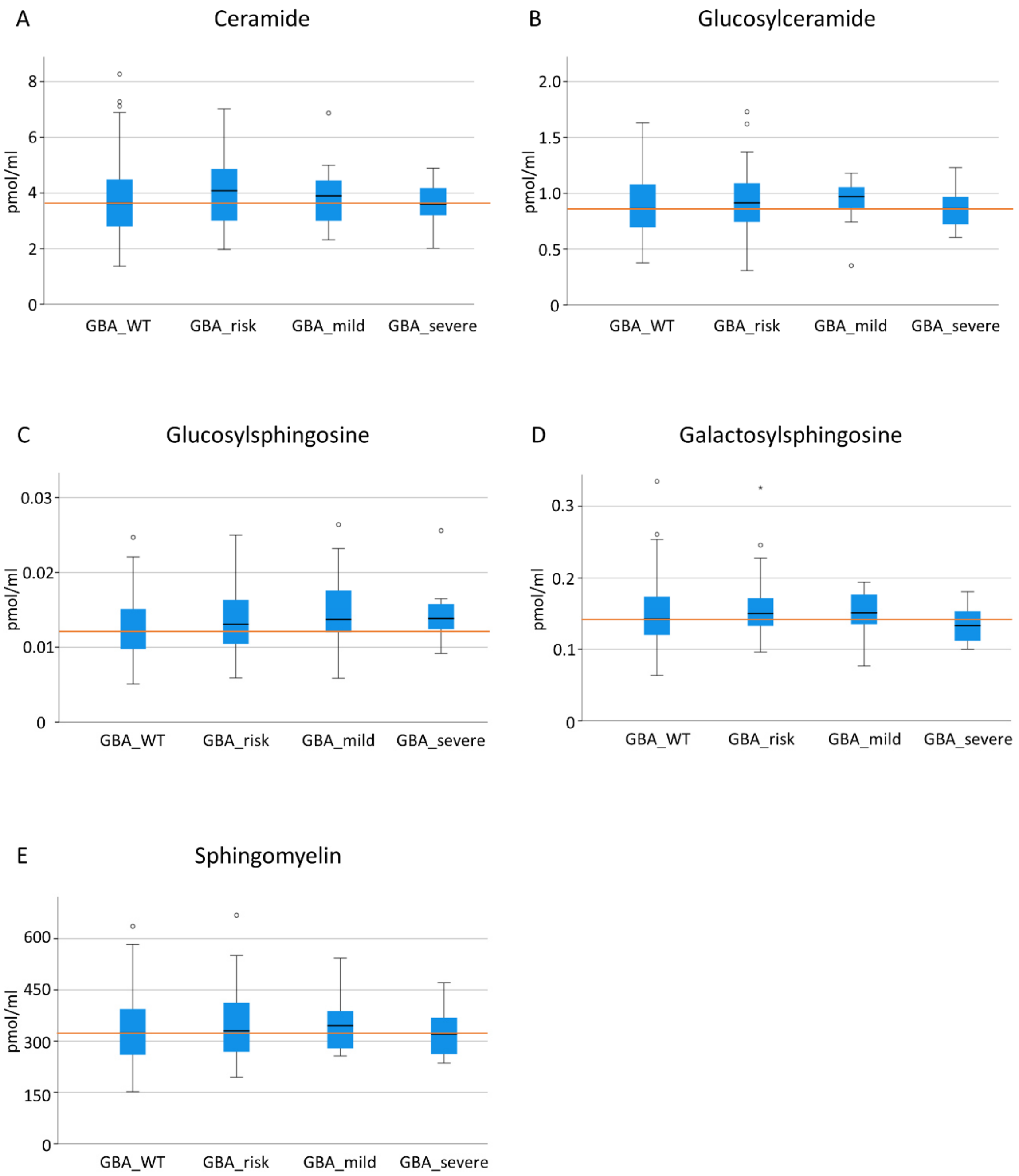

Supplemental Figure 2: Kaplan-Meier curves depicting the duration until development of cognitive impairment in PD_{GBA_WT}+PD_{GBA} stratified by sphingolipid tertiles.

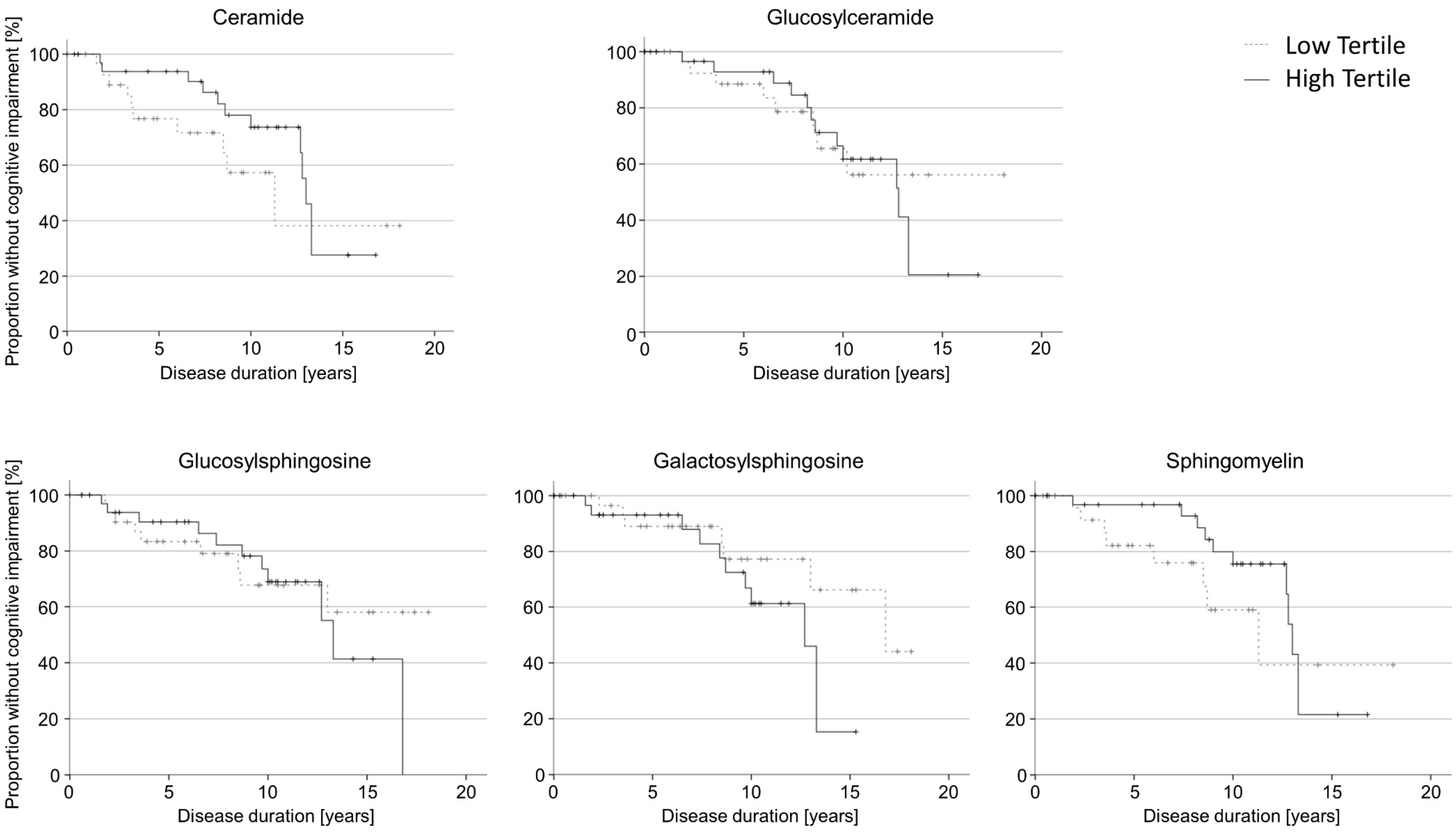

Supplemental Table 1: Demographics and clinical data

	HC n=40	PD_{GBA_WT} n=79	PD_{GBA} n=73	DLB_{GBA_WT} n=26	DLB_{GBA} n=11	$PD_{Gaucher}$ n=5	p-value
Sex, male % (n)	60 (24)	65 (51)	67 (49)	77 (20)	100 (11)	100 (5)	0.071
Age, years	55 ± 15	63 ± 11***	63 ± 9***	72 ± 5$^{***,\#\#\#,§§§}$	69 ± 6$^{***,\#,§}$	65 ± 12*	<0.001
Age at onset, years	-	57 ± 10	57 ± 9	68 ± 6$^{\#\#\#,§§§}$	66 ± 6$^{\#\#,§§}$	57 ± 18$^{°}$	<0.001
Disease duration, years	-	5 ± 6	6 ± 6	3 ± 2	3 ± 2	9 ± 7	0.165
Hoehn&Yahr	-	2.1 ± 0.7	2.1 ± 0.6	2.4 ± 0.6§	2.3 ± 0.4	2.8 ± 0.5	0.576^{a}
UPDRS-III	-	26 ± 12 (n=67)	29 ± 12 (n=64)	28 ± 7 (n=7)	29 ± 11 (n=6)	24 ± 21	0.770^{a}
MoCA	27 ± 3 (n=12)	25 ± 5 (n=69)	25 ± 4 (n=65)	13 ± 5$^{***,\#\#\#,§§§}$ (n=22)	15 ± 7$^{***,\#\#\#,§§§}$	21 ± 7$^{*,°°°,+}$	<0.001^{a}

Data are shown as mean ± standard deviation. ^{a} ANCOVA with age at co-variable; * versus HC, # versus PD_{GBA_WT}; § versus PD_{GBA}, ° versus DLB_{GBA_WT},$^{+}$ versus DLB_{GBA}

*p<0.05; **p<0.01; ***p≤0.001

DLB; Dementia with Lewy Bodies; GBA, Glucocerebrosidase; HC, healthy control; MoCA, Montreal Cognitive Assessment; PD, Parkinson's Disease; UPDRS-III, Unified Parkinson's Disease rating Scale part III; WT, wildtype

Supplemental Table 2: Overview of genetic variants.

<table>
<tr><th></th><th>GBA_risk</th><th>GBA_mild</th><th>GBA_severe</th><th>Other gene variants</th></tr>
<tr><td>HC (n=0/40)</td><td>-</td><td>-</td><td>-</td><td>-</td></tr>
<tr><td>DLB (n=11/37)</td><td>3x p.E365K
2x p.T408M</td><td>2x p.N409S</td><td>1x p.L483P
1x p.R159Q
1x Exon3+4 Recombination</td><td>1x p.E365K (GBA1) + p.437L (PRKN)</td></tr>
<tr><td>PD (n=71/152)</td><td>31x p.E365K
14x p.T408M</td><td>11x p.N409S
1x p.S310G
1x p.D179H + p.E365K</td><td>10x p.L483P
1x p.R398*
1x p.W218*
1x p.W223R
1x p.Y244C
1x c.11511G>A</td><td>-</td></tr>
<tr><td>PD_Gaucher
n=5</td><td colspan="3">p.N409S / p.S310G
p.L483R / p.N409S
p.L483P / p.N409S
p.R202* / p.N409S
c.1265-1319del / p.483P + p.D448H</td><td>-</td></tr>
</table>

GluSph (d18:1) and GalSph (d18:1) measurements with example Chromatograms

GlcSph (d18:1) and GalSph (d18:1) and spiked internal standards GlcSph (d18:1)-d5 and GalSph (d18:1)-d5 were isolated from 200 µL of human CSF by solid phase extraction (SPE) using 1cc 30 mg Waters Oasis MCX cartridges (Waters). In short, cartridges were equilibrated with 1 mL methanol (MeOH) and 1 mL water, after which the acidified CSF samples (0.2 mL CSF + 0.1 mL 1.0/2.0 mg/mL bovine serum albumin/ascorbic acid in phosphate buffer saline (PBS) + 0.75 mL 1% H3PO4 + 0.1 mL 2.0 mg/mL ascorbic acid in MeOH) were loaded on the cartridges. Sample were washed with 2 x 0.75 mL water and 2 x 0.75 mL MeOH after which the analytes were eluted with 2 x 0.4 mL 1% NH4FA in MeOH in a tube contain 0.1 mL of 2mg/mL ascorbic acid in MeOH.

After evaporation, extracts were redissolved in 0.0500 mL of 2 mg/mL ascorbic acid in chloroform:MeOH : UPW (5:1:0.1 (v/v/v)) after which 0.100 mL ACN was added. Blank-, calibration-, QC- and study samples were injected into the Chromatographic system (Shimadzu) on a Hypersil GOLD Silica column (100 x 2.1mm (length x internal diameter), 1.9 µm (particle size)), Thermo Fisher Scientific) using a gradient elution with 1M NH4FA : FA : water : ACN (20 : 6 : 40 : 2000 (v/v/v/v) as mobile phase A and 1M NH4FA : water : ACN (5 : 500 : 10 (v/v/v) as mobile phase B. An API6500 tandem mass spectrometer (Sciex) equipped with a Turbo Ion Spray probe operating in the positive multiple reaction monitoring mode was used for quantification. MS transitions were 462 → 282 and 467 → 287 (unit resolution) for GlcSph (d18:1)/GalSph(d18:1) and the internal standards GlcSph(d18:1)-d5/GalSph(d18:1)-d5, respectively. GlcSph(d18:1) and GalSph(d18:1) were chromatographically separated since the molecules are isomers and share similar fragments.

The analytical ranges were 0.00500 – 1.00 pmol/mL for both analytes. Each analytical run included duplicate QC samples at three levels (QC-Low at 0.0300 pmol/mL, QC-Medium at 0.150 pmol/mL and QC-High at 0.800 pmol/mL for both analytes) and a representative QC-Pool sample in quadruplicate.

Data shown below has examples from a single run for the standard curve, blank, double blank, quality control samples, and examples of samples analyzed in this study.

Blank and Double Blank samples:

1. Double blank (no analyte or internal standard IS)
2. Single blank (with internal standard IS)

Sample Name	DOUBLE BLANK	Sample Type	Double Blank
Sample ID	1700763-847131	Dilution	1.00
File Name	21287-AB011\21287-AB011.wiff	Acquisition date	2021-12-22 17:13:34

GALSPH(D18:1) (N/A pmol/mL; Area 0.00e+000 cps; RT 0.00 min)

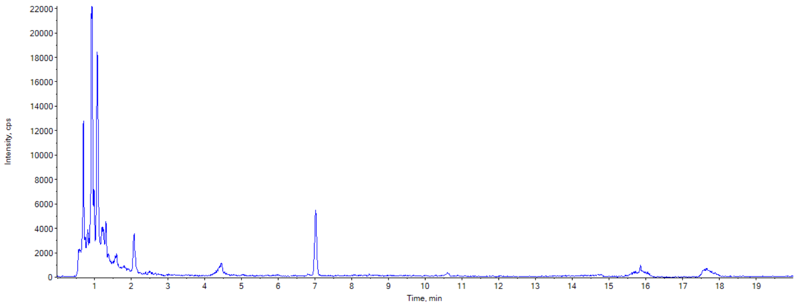

462.200/282.300 Da

ZOOMED

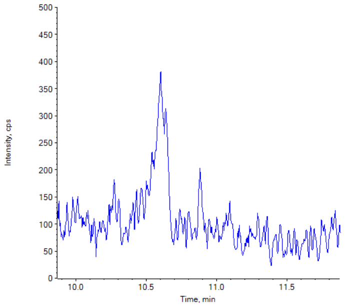

GLUSPH(D18:1) (N/A pmol/mL; Area 1.32e+003 cps; RT 10.6 min)

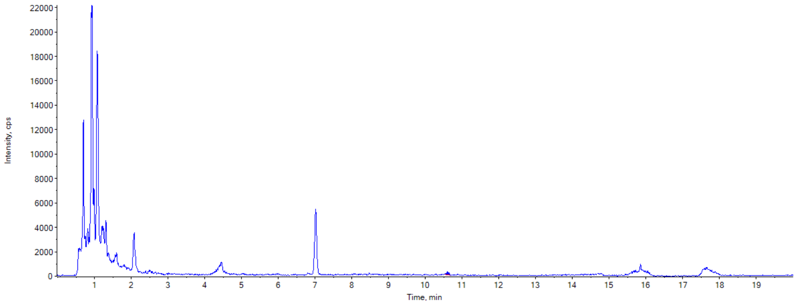

462.200/282.300 Da

ZOOMED

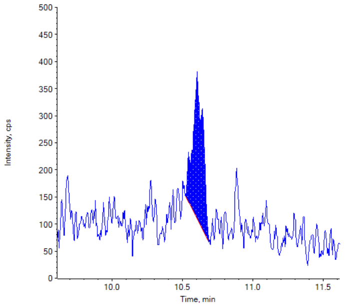

IS_GALSPH(D18:1) (Area 0.00e+000 cps; RT 0.00 min; 467.300/287.300 Da)

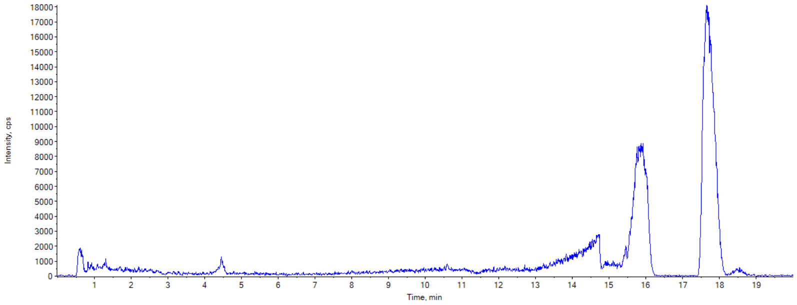

467.300/287.300 Da

ZOOMED

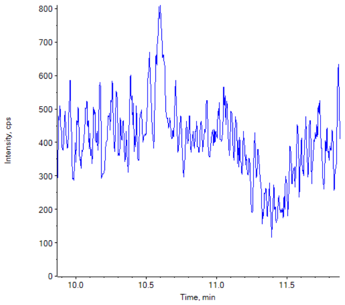

IS_GLUSPH(D18:1) (Area 0.00e+000 cps; RT 0.00 min; 467.300/287.300 Da)

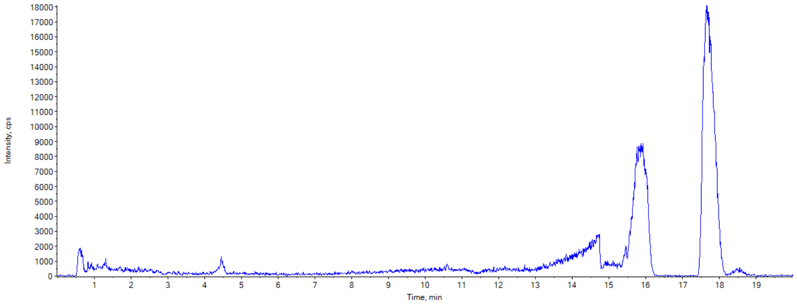

467.300/287.300 Da

ZOOMED

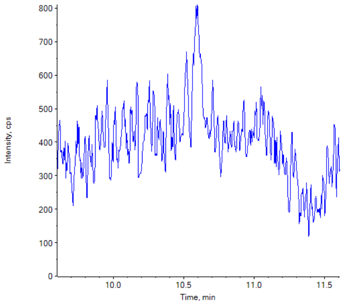

Sample Name	BLANK	Sample Type	Blank
Sample ID	1700764-847132	Dilution	1.00
File Name	21287-AB011\21287-AB011.wiff	Acquisition date	2021-12-22 17:44:07

GALSPH(D18:1) (N/A pmol/mL; Area 0.00e+000 cps; RT 0.00 min)

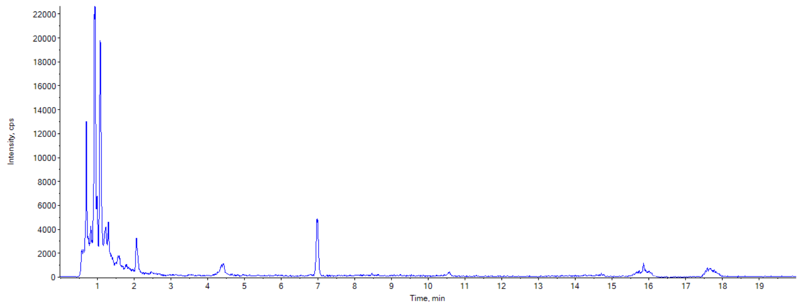

462.200/282.300 Da

ZOOMED

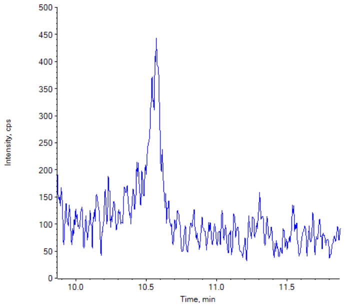

GLUSPH(D18:1) (N/A pmol/mL; Area 7.92e+002 cps; RT 10.6 min)

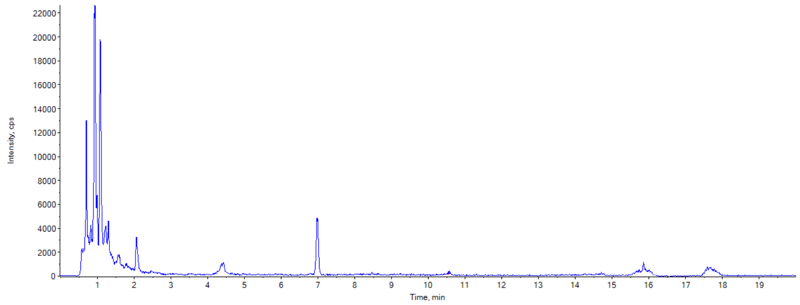

462.200/282.300 Da

ZOOMED

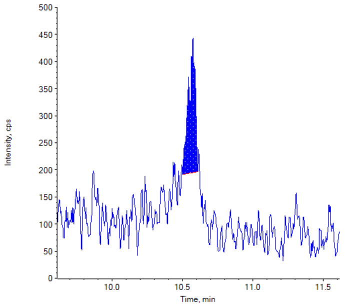

IS_GALSPH(D18:1) (Area 2.53e+005 cps; RT 10.8 min; 467.300/287.300 Da)

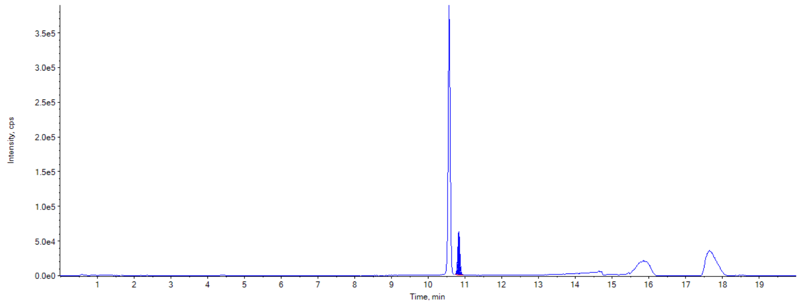

467.300/287.300 Da

ZOOMED

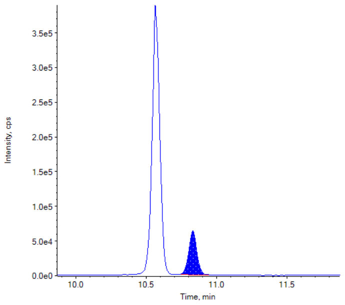

IS_GLUSPH(D18:1) (Area 1.45e+006 cps; RT 10.6 min; 467.300/287.300 Da)

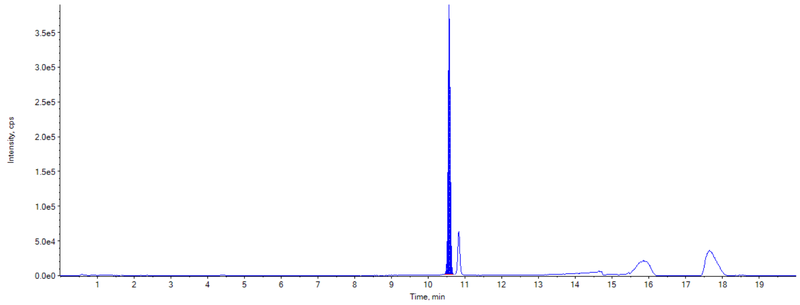

467.300/287.300 Da

ZOOMED

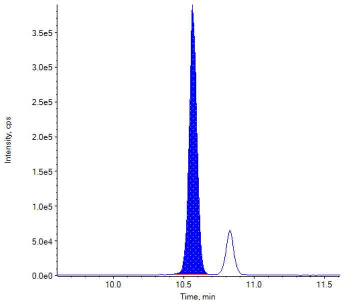

Standard curve (GluSph & GalSph):

1. 0.005 pmol/ml
2. 0.01 pmol/ml
3. 0.02 pmol/ml
4. 0.04 pmol/ml
5. 0.1 pmol/ml
6. 0.2 pmol/ml
7. 0.4 pmol/ml
8. 0.8 pmol/ml
9. 1.0 pmol/ml

Sample Name	ST-13088_ST-01	Sample Type	Standard
Sample ID	1700765-847133	Dilution	1.00
File Name	21287-AB011\21287-AB011.wiff	Acquisition date	2021-12-22 18:14:38

GALSPH(D18:1) (0.00501 pmol/mL; Area 2.69e+003 cps; RT 10.9 min)

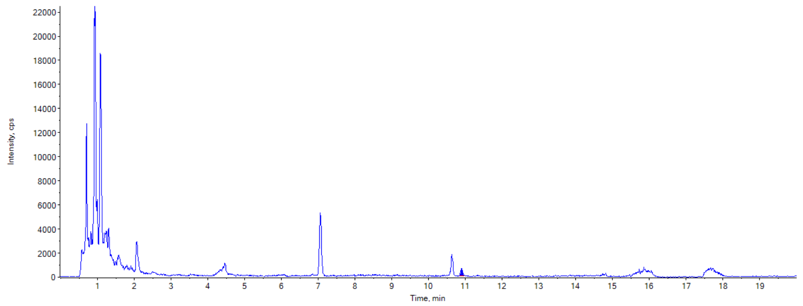

462.200/282.300 Da

ZOOMED

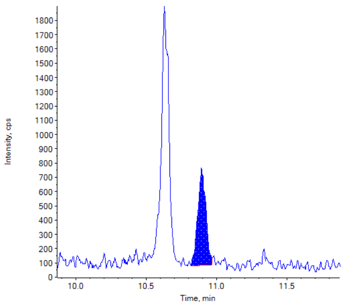

GLUSPH(D18:1) (0.00496 pmol/mL; Area 6.06e+003 cps; RT 10.6 min)

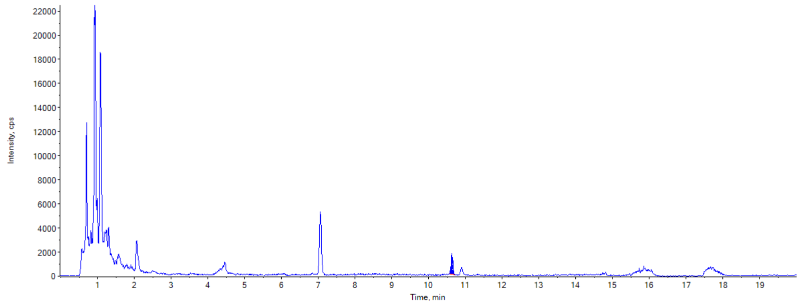

462.200/282.300 Da

ZOOMED

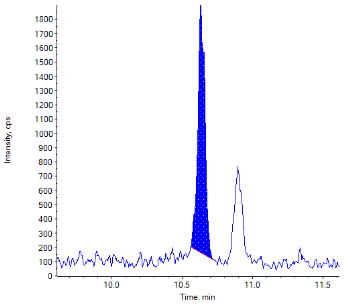

IS_GALSPH(D18:1) (Area 2.66e+005 cps; RT 10.9 min; 467.300/287.300 Da)

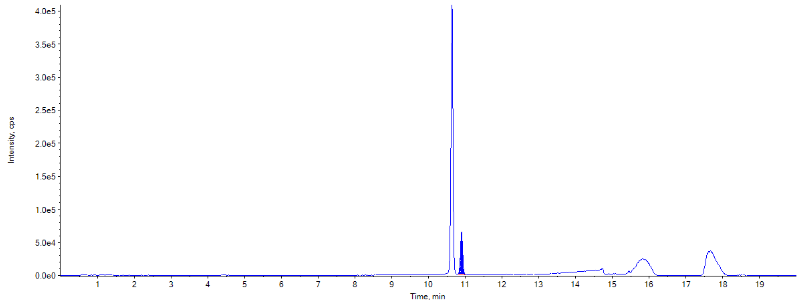

467.300/287.300 Da

ZOOMED

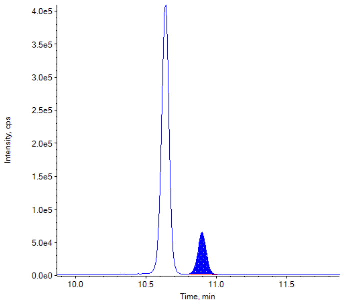

IS_GLUSPH(D18:1) (Area 1.55e+006 cps; RT 10.6 min; 467.300/287.300 Da)

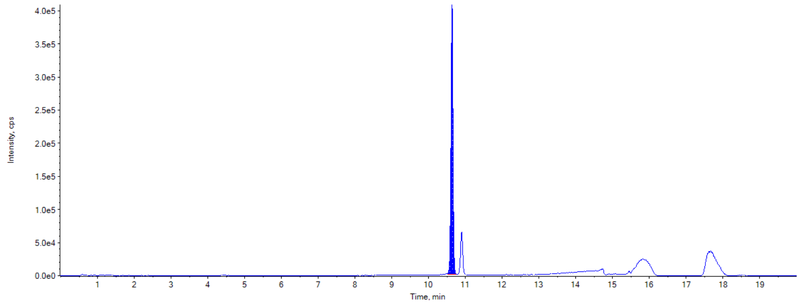

467.300/287.300 Da

ZOOMED

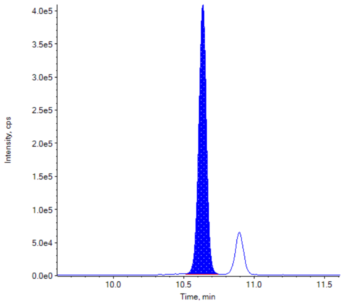

Sample Name	ST-13089_ST-02	Sample Type	Standard
Sample ID	1700766-847134	Dilution	1.00
File Name	21287-AB011\21287-AB011.wiff	Acquisition date	2021-12-22 18:45:11

GALSPH(D18:1) (0.00993 pmol/mL; Area 5.04e+003 cps; RT 10.9 min)

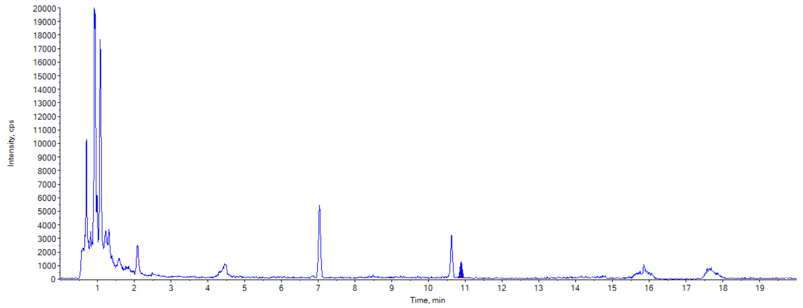

462.200/282.300 Da

ZOOMED

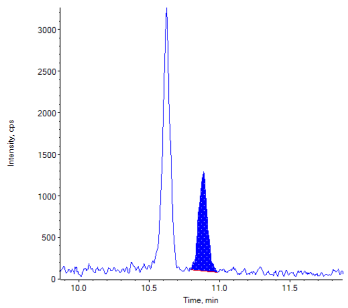

GLUSPH(D18:1) (0.0103 pmol/mL; Area 1.09e+004 cps; RT 10.6 min)

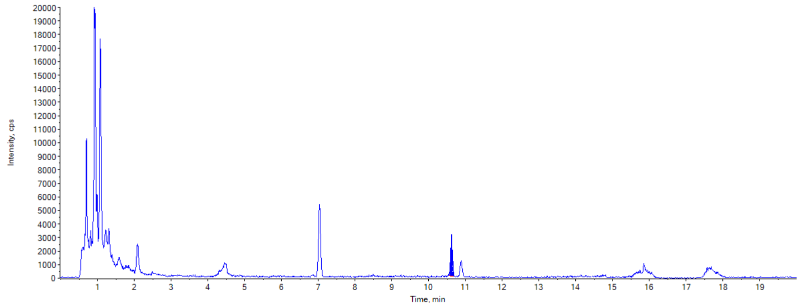

462.200/282.300 Da

ZOOMED

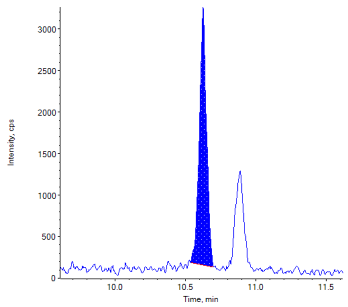

IS_GALSPH(D18:1) (Area 2.53e+005 cps; RT 10.9 min; 467.300/287.300 Da)

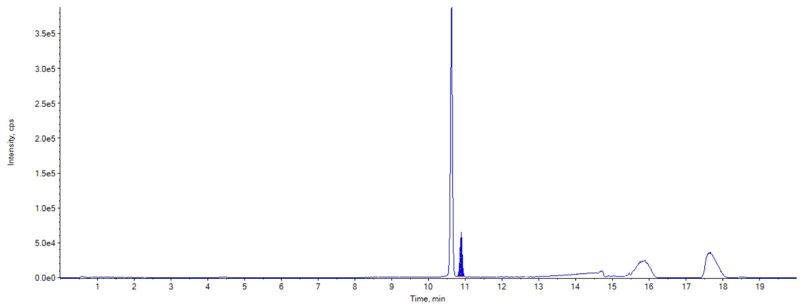

467.300/287.300 Da

ZOOMED

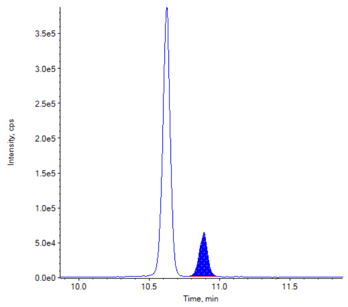

IS_GLUSPH(D18:1) (Area 1.44e+006 cps; RT 10.6 min; 467.300/287.300 Da)

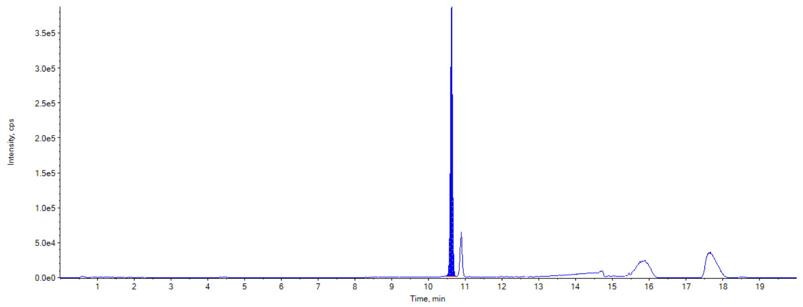

467.300/287.300 Da

ZOOMED

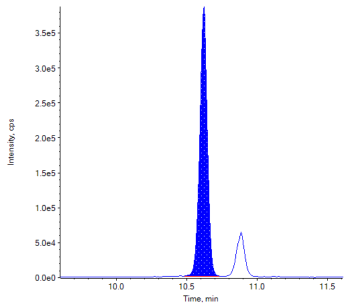

Sample Name	ST-13090_ST-03	Sample Type	Standard
Sample ID	1700767-847135	Dilution	1.00
File Name	21287-AB011\21287-AB011.wiff	Acquisition date	2021-12-22 19:15:45

GALSPH(D18:1) (0.0201 pmol/mL; Area 9.86e+003 cps; RT 10.9 min)

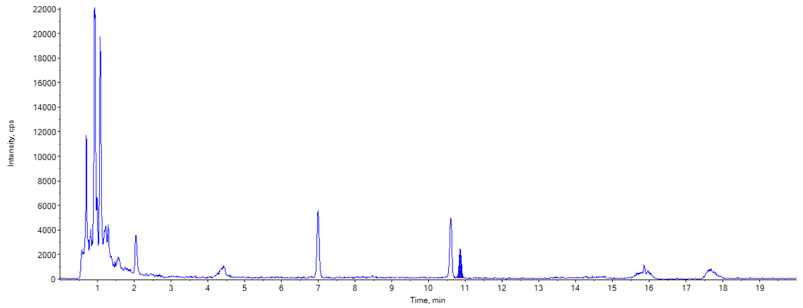

462.200/282.300 Da

ZOOMED

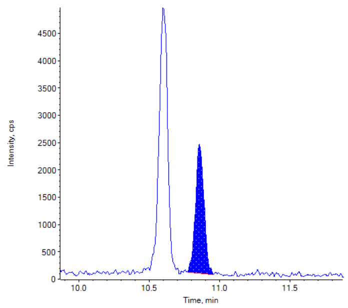

GLUSPH(D18:1) (0.0192 pmol/mL; Area 2.00e+004 cps; RT 10.6 min)

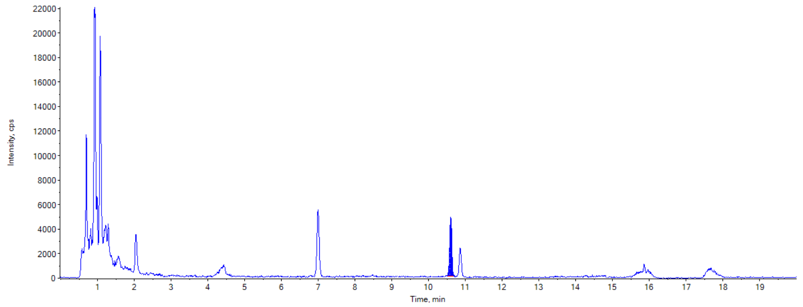

462.200/282.300 Da

ZOOMED

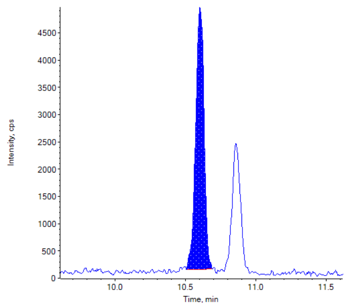

IS_GALSPH(D18:1) (Area 2.46e+005 cps; RT 10.9 min; 467.300/287.300 Da)

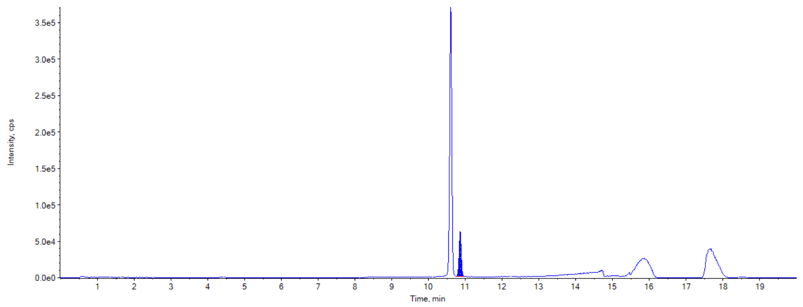

467.300/287.300 Da

ZOOMED

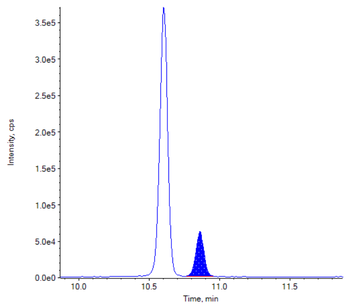

IS_GLUSPH(D18:1) (Area 1.45e+006 cps; RT 10.6 min; 467.300/287.300 Da)

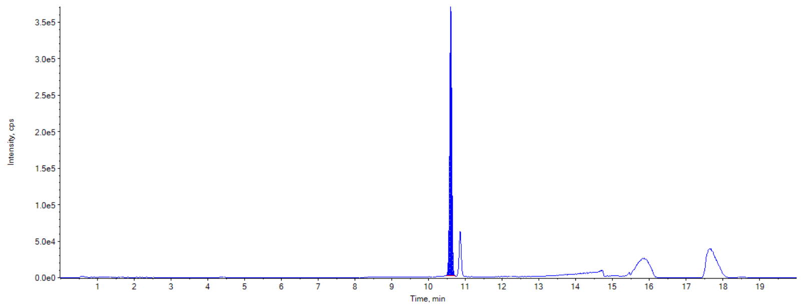

467.300/287.300 Da

ZOOMED

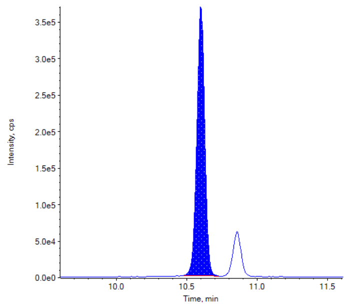

Sample Name	ST-13091_ST-04	Sample Type	Standard
Sample ID	1700768-847136	Dilution	1.00
File Name	21287-AB011\21287-AB011.wiff	Acquisition date	2021-12-22 19:46:19

GALSPH(D18:1) (0.0406 pmol/mL; Area 2.21e+004 cps; RT 10.9 min)

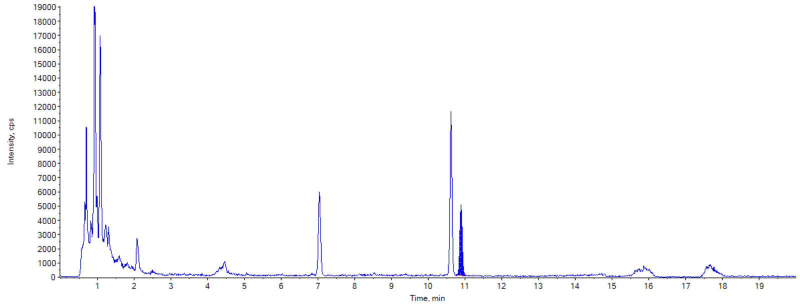

462.200/282.300 Da

ZOOMED

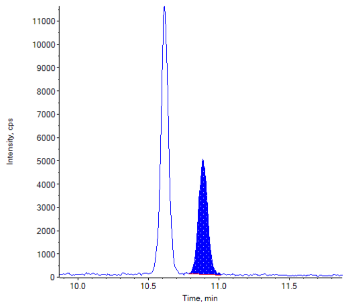

GLUSPH(D18:1) (0.0414 pmol/mL; Area 4.30e+004 cps; RT 10.6 min)

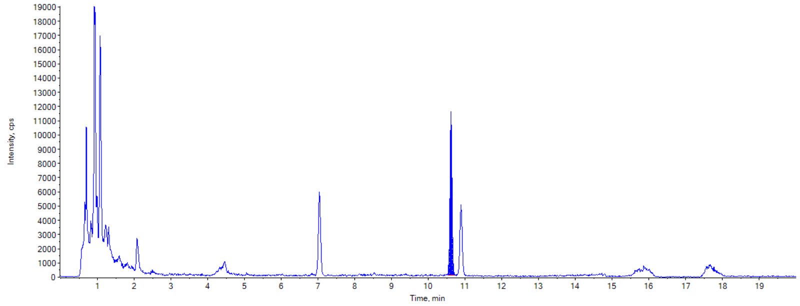

462.200/282.300 Da

ZOOMED

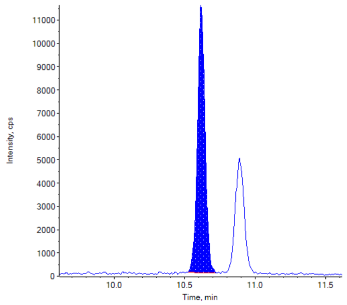

IS_GALSPH(D18:1) (Area 2.73e+005 cps; RT 10.9 min; 467.300/287.300 Da)

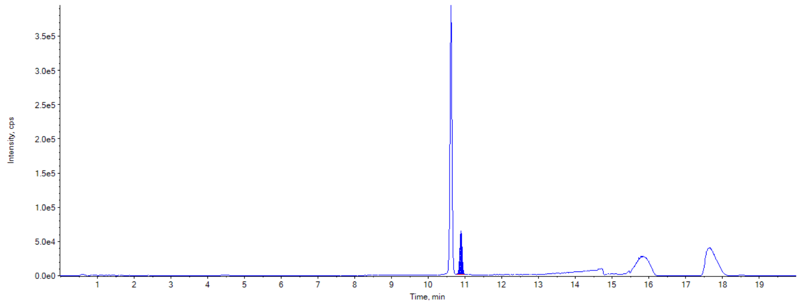

467.300/287.300 Da

ZOOMED

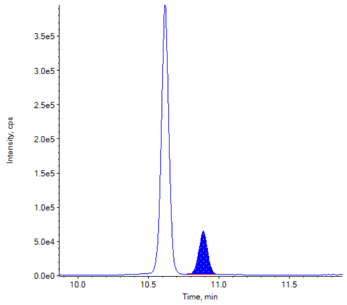

IS_GLUSPH(D18:1) (Area 1.47e+006 cps; RT 10.6 min; 467.300/287.300 Da)

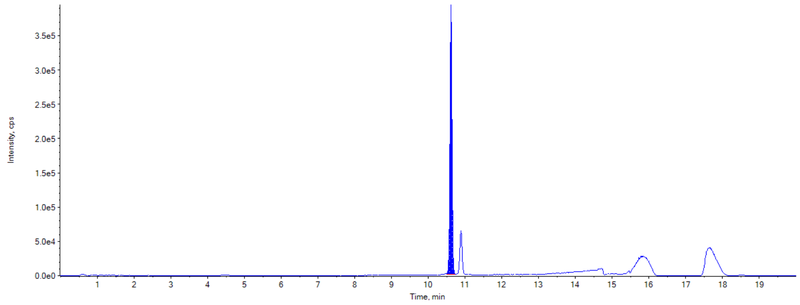

467.300/287.300 Da

ZOOMED

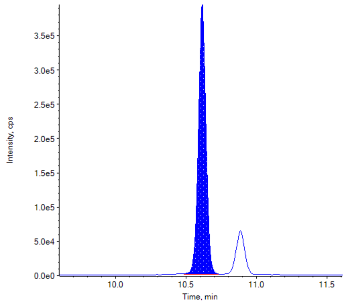

Sample Name	ST-13092_ST-05	Sample Type	Standard
Sample ID	1700769-847137	Dilution	1.00
File Name	21287-AB011\21287-AB011.wiff	Acquisition date	2021-12-22 20:16:54

GALSPH(D18:1) (0.100 pmol/mL; Area 5.43e+004 cps; RT 10.9 min)

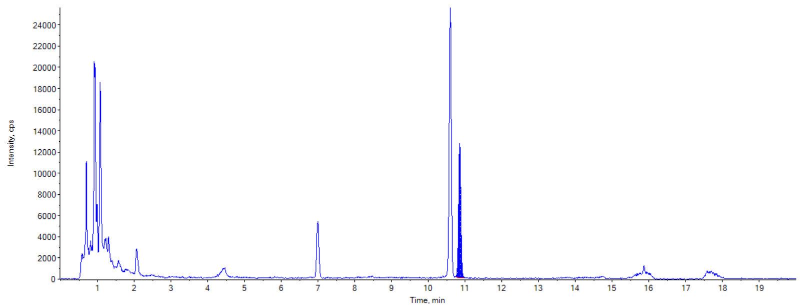

462.200/282.300 Da

ZOOMED

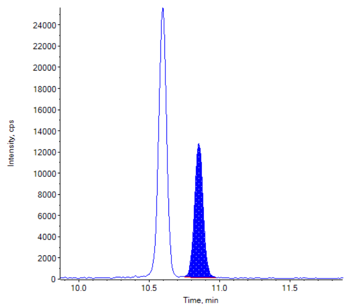

GLUSPH(D18:1) (0.0997 pmol/mL; Area 1.01e+005 cps; RT 10.6 min)

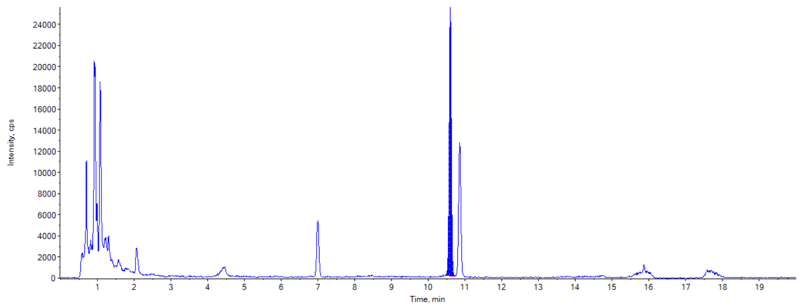

462.200/282.300 Da

ZOOMED

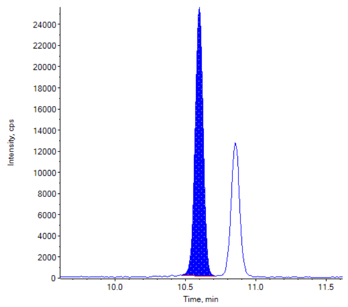

IS_GALSPH(D18:1) (Area 2.72e+005 cps; RT 10.9 min; 467.300/287.300 Da)

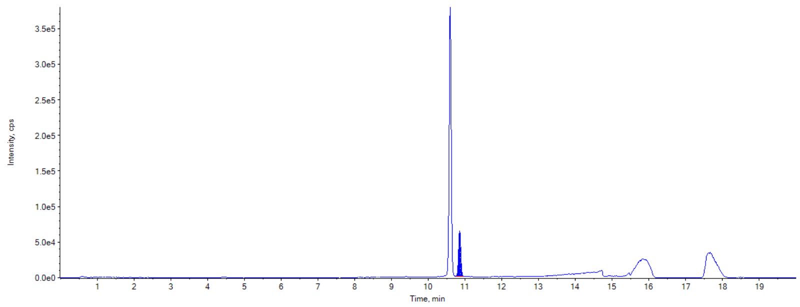

467.300/287.300 Da

ZOOMED

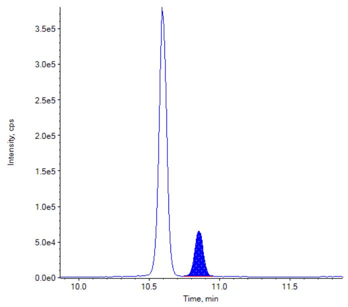

IS_GLUSPH(D18:1) (Area 1.44e+006 cps; RT 10.6 min; 467.300/287.300 Da)

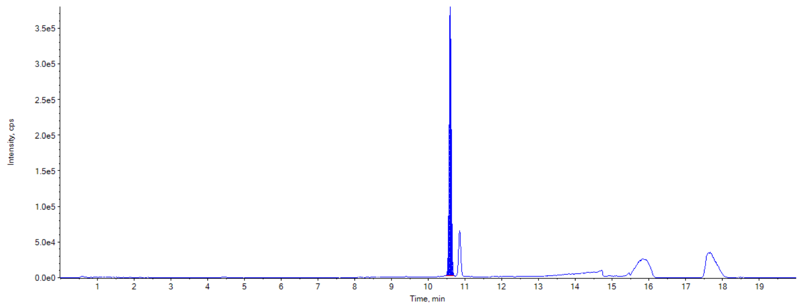

467.300/287.300 Da

ZOOMED

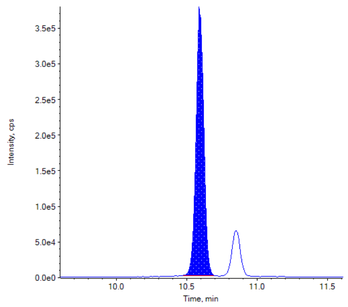

Sample Name	ST-13093_ST-06	Sample Type	Standard
Sample ID	1700770-847138	Dilution	1.00
File Name	21287-AB011\21287-AB011.wiff	Acquisition date	2021-12-22 20:47:28

GALSPH(D18:1) (0.195 pmol/mL; Area 1.02e+005 cps; RT 10.8 min)

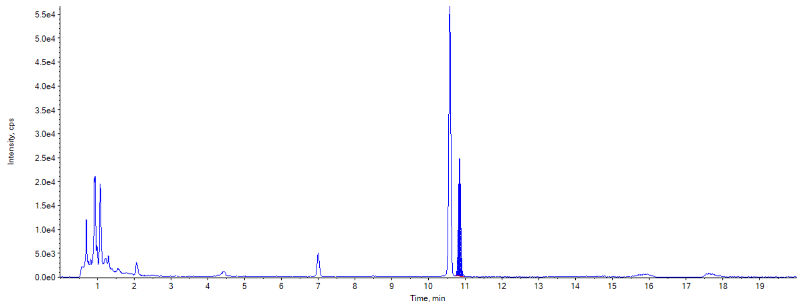

462.200/282.300 Da

ZOOMED

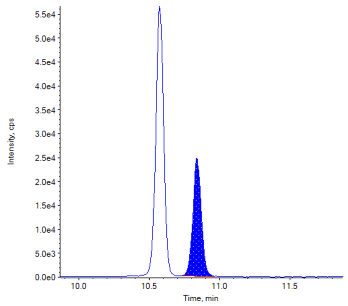

GLUSPH(D18:1) (0.195 pmol/mL; Area 2.21e+005 cps; RT 10.6 min)

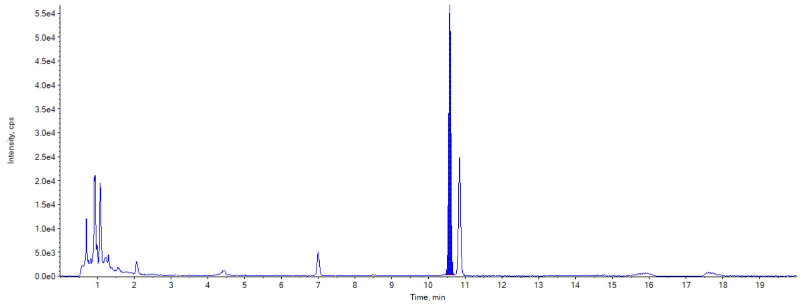

462.200/282.300 Da

ZOOMED

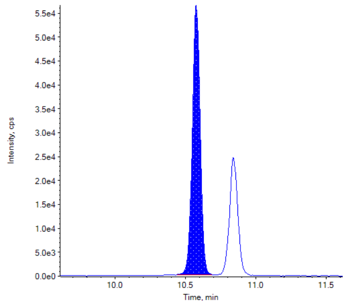

IS_GALSPH(D18:1) (Area 2.63e+005 cps; RT 10.8 min; 467.300/287.300 Da)

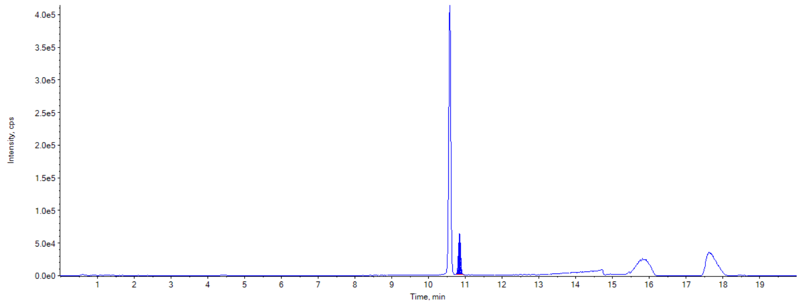

467.300/287.300 Da

ZOOMED

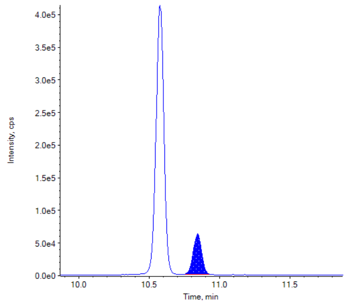

IS_GLUSPH(D18:1) (Area 1.62e+006 cps; RT 10.6 min; 467.300/287.300 Da)

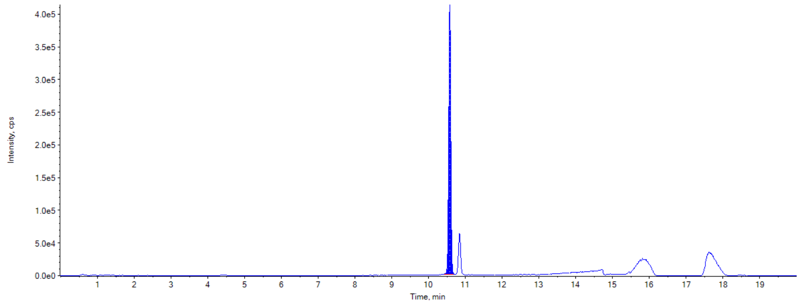

467.300/287.300 Da

ZOOMED

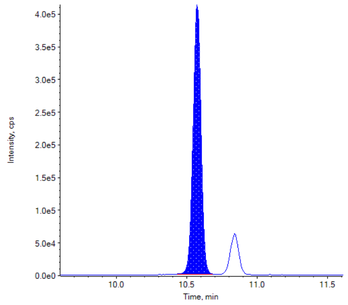

Sample Name	ST-13094_ST-07	Sample Type	Standard
Sample ID	1700771-847139	Dilution	1.00
File Name	21287-AB011\21287-AB011.wiff	Acquisition date	2021-12-22 21:18:00

GALSPH(D18:1) (0.404 pmol/mL; Area 2.05e+005 cps; RT 10.9 min)

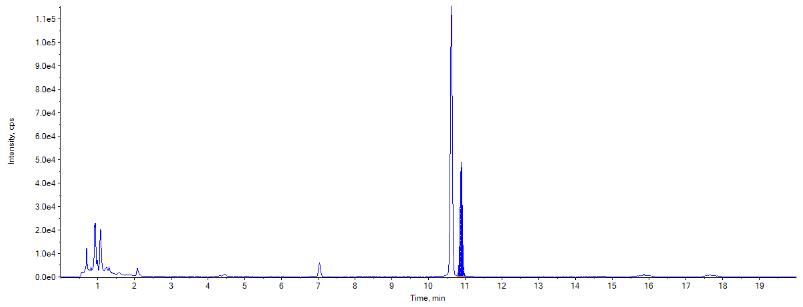

462.200/282.300 Da

ZOOMED

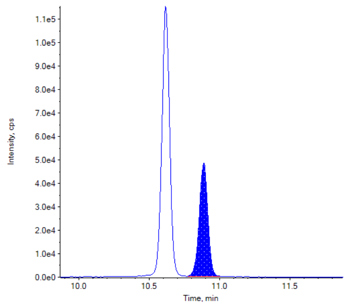

GLUSPH(D18:1) (0.404 pmol/mL; Area 4.43e+005 cps; RT 10.6 min)

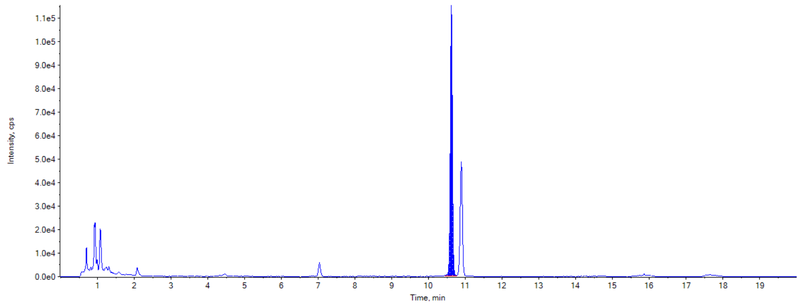

462.200/282.300 Da

ZOOMED

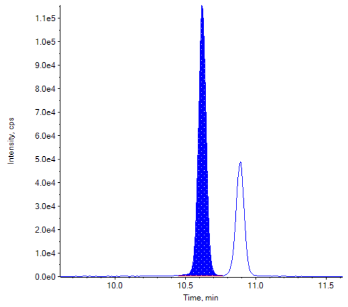

IS_GALSPH(D18:1) (Area 2.55e+005 cps; RT 10.9 min; 467.300/287.300 Da)

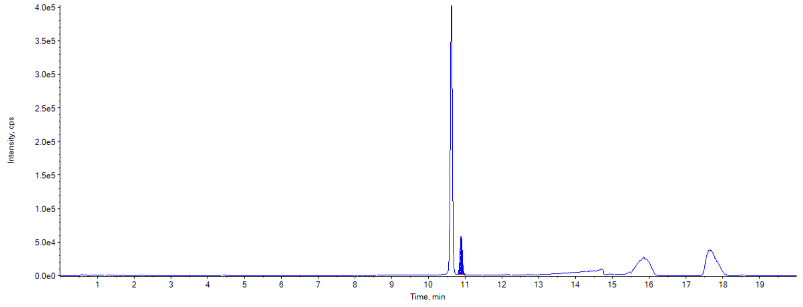

467.300/287.300 Da

ZOOMED

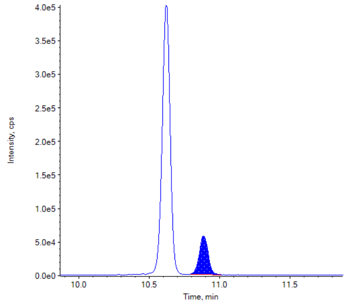

IS_GLUSPH(D18:1) (Area 1.57e+006 cps; RT 10.6 min; 467.300/287.300 Da)

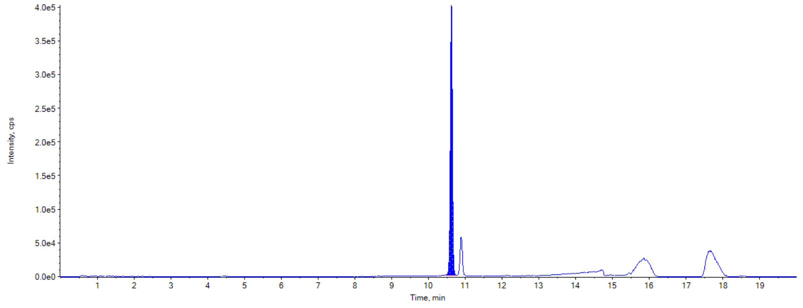

467.300/287.300 Da

ZOOMED

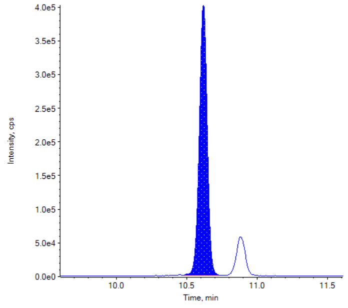

Sample Name	ST-13095_ST-08	Sample Type	Standard
Sample ID	1700772-847140	Dilution	1.00
File Name	21287-AB011\21287-AB011.wiff	Acquisition date	2021-12-22 21:48:33

GALSPH(D18:1) (0.804 pmol/mL; Area 3.76e+005 cps; RT 10.9 min)

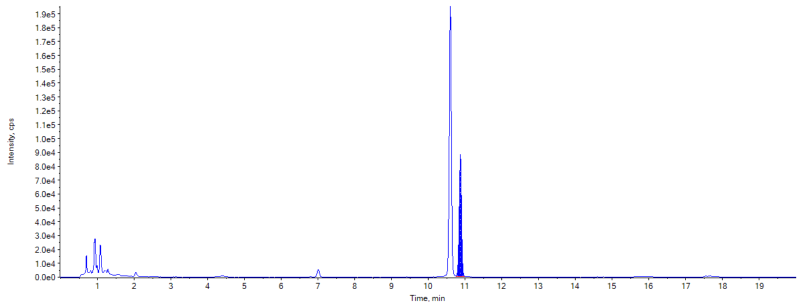

462.200/282.300 Da

ZOOMED

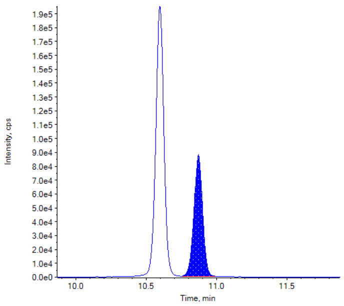

GLUSPH(D18:1) (0.800 pmol/mL; Area 7.72e+005 cps; RT 10.6 min)

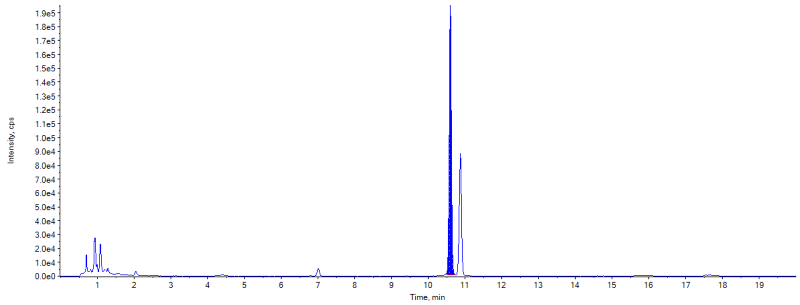

462.200/282.300 Da

ZOOMED

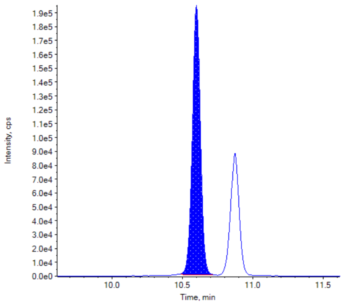

IS_GALSPH(D18:1) (Area 2.34e+005 cps; RT 10.9 min; 467.300/287.300 Da)

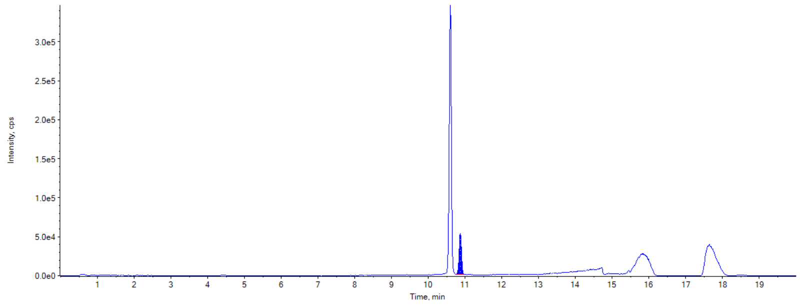

467.300/287.300 Da

ZOOMED

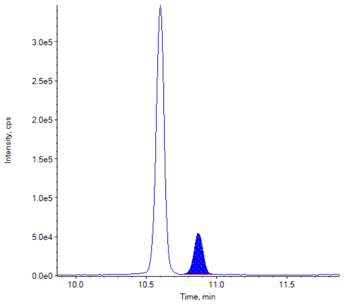

IS_GLUSPH(D18:1) (Area 1.38e+006 cps; RT 10.6 min; 467.300/287.300 Da)

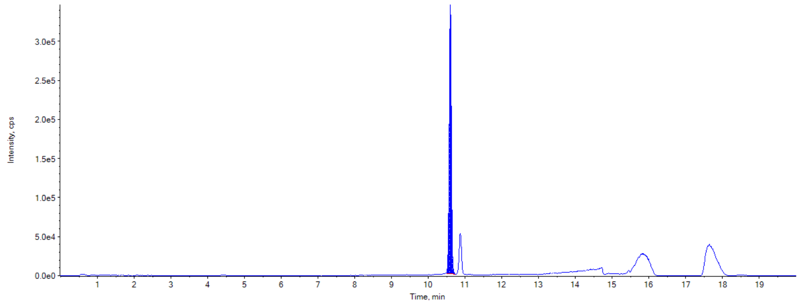

467.300/287.300 Da

ZOOMED

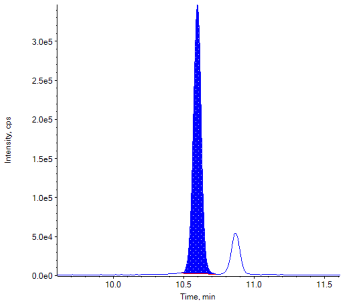

Sample Name	ST-13096_ST-09	Sample Type	Standard
Sample ID	1700773-847141	Dilution	1.00
File Name	21287-AB011\21287-AB011.wiff	Acquisition date	2021-12-22 22:19:09

GALSPH(D18:1) (0.996 pmol/mL; Area 4.78e+005 cps; RT 10.9 min)

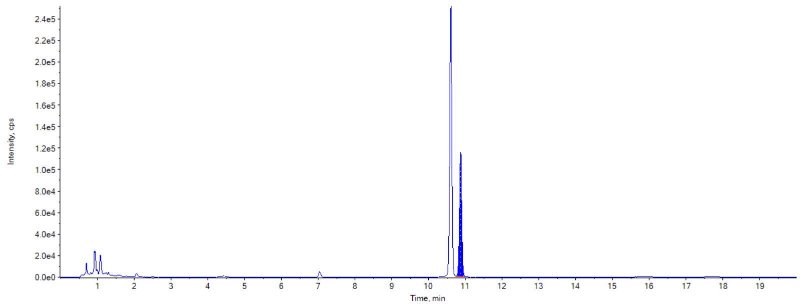

462.200/282.300 Da

ZOOMED

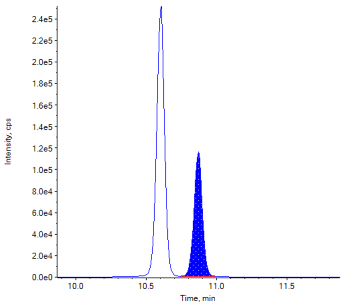

GLUSPH(D18:1) (1.00 pmol/mL; Area 9.81e+005 cps; RT 10.6 min)

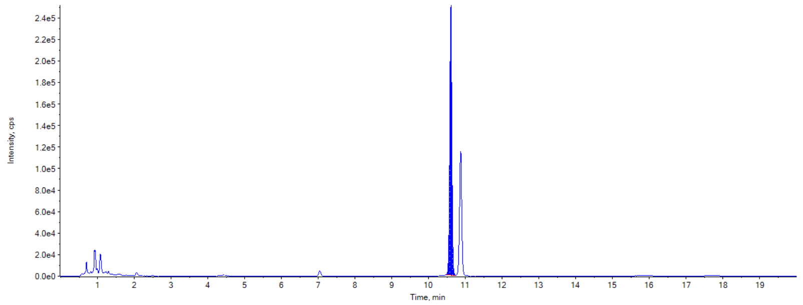

462.200/282.300 Da

ZOOMED

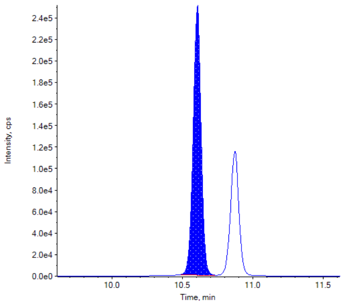

IS_GALSPH(D18:1) (Area 2.41e+005 cps; RT 10.9 min; 467.300/287.300 Da)

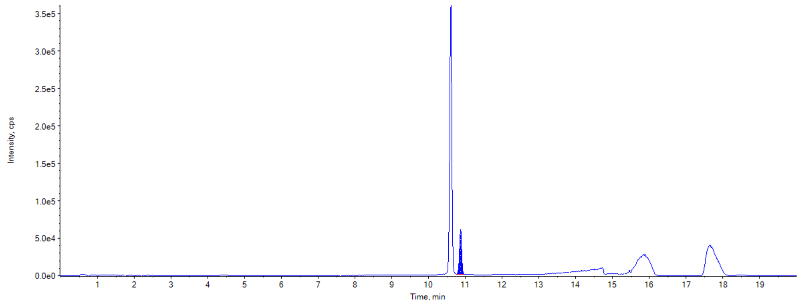

467.300/287.300 Da

ZOOMED

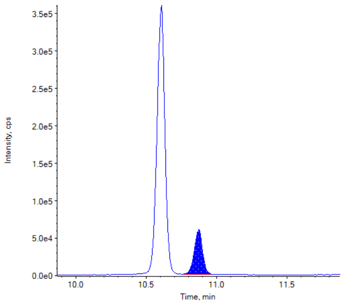

IS_GLUSPH(D18:1) (Area 1.40e+006 cps; RT 10.6 min; 467.300/287.300 Da)

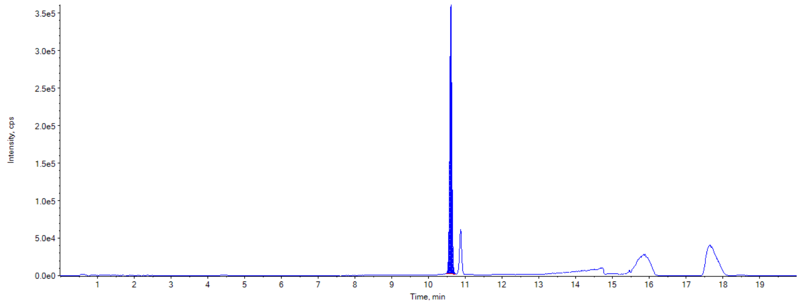

467.300/287.300 Da

ZOOMED

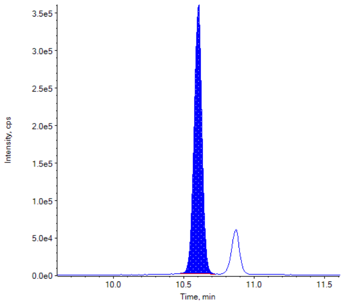

Quality control samples for both analytes together:

1. QC-High at 0.800 pmol/mL
2. QC-Medium at 0.150 pmol/mL
3. QC-Low at 0.0300 pmol/mL
4. Pooled QC sample (CSF)

Sample Name	Q14327-011_QC-H	Sample Type	Quality Control
Sample ID	1700727-847122	Dilution	1.00
File Name	21287-AB011\21287-AB011.wiff	Acquisition date	2021-12-22 22:49:42

GALSPH(D18:1) (0.812 pmol/mL; Area 3.64e+005 cps; RT 10.9 min)

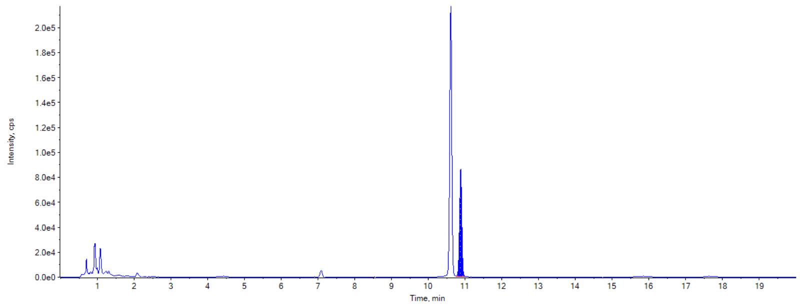

462.200/282.300 Da

ZOOMED

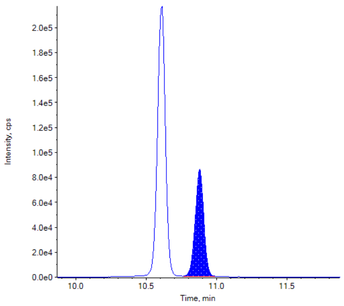

GLUSPH(D18:1) (0.789 pmol/mL; Area 8.50e+005 cps; RT 10.6 min)

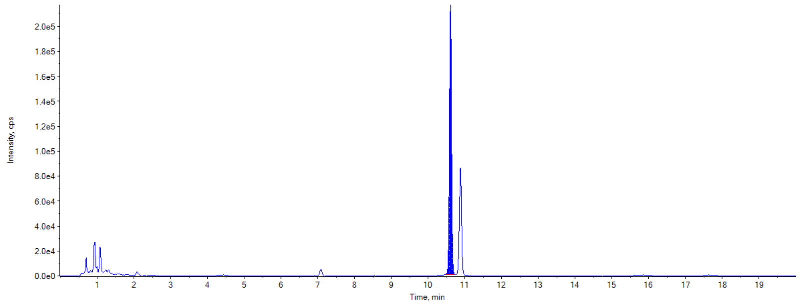

462.200/282.300 Da

ZOOMED

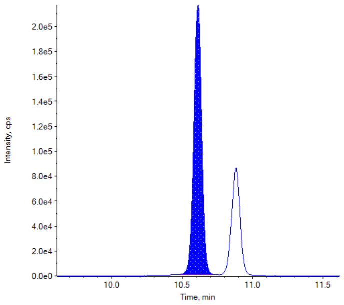

IS_GALSPH(D18:1) (Area 2.25e+005 cps; RT 10.9 min; 467.300/287.300 Da)

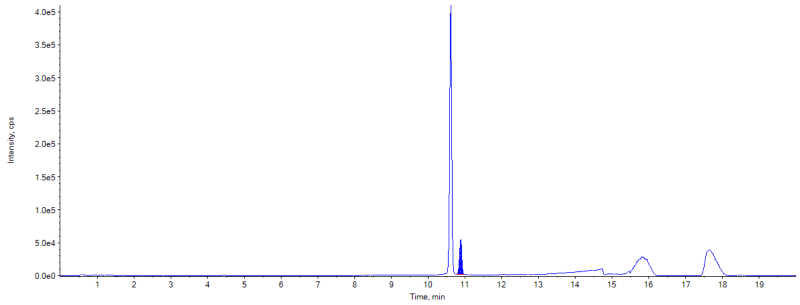

467.300/287.300 Da

ZOOMED

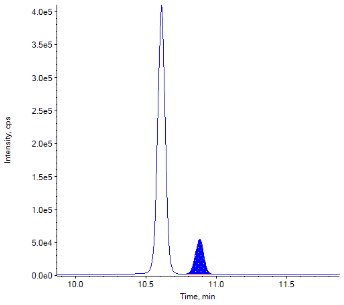

IS_GLUSPH(D18:1) (Area 1.55e+006 cps; RT 10.6 min; 467.300/287.300 Da)

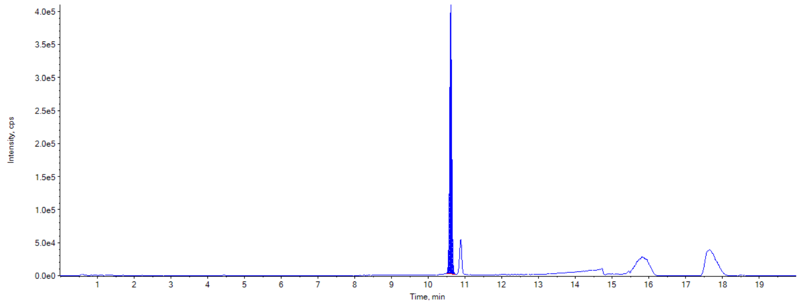

467.300/287.300 Da

ZOOMED

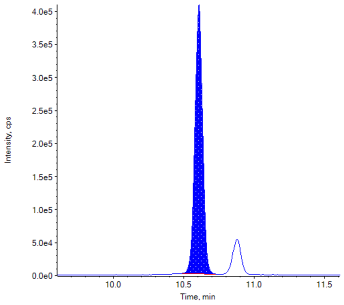

Sample Name	Q14328-011_QC-M	Sample Type	Quality Control
Sample ID	1700737-847124	Dilution	1.00
File Name	21287-AB011\21287-AB011.wiff	Acquisition date	2021-12-22 23:20:16

GALSPH(D18:1) (0.155 pmol/mL; Area 8.70e+004 cps; RT 10.9 min)

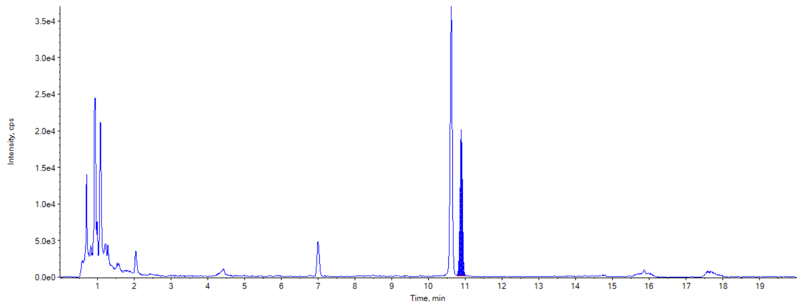

462.200/282.300 Da

ZOOMED

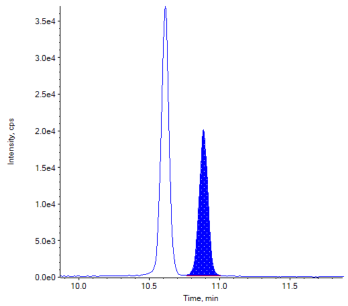

GLUSPH(D18:1) (0.145 pmol/mL; Area 1.44e+005 cps; RT 10.6 min)

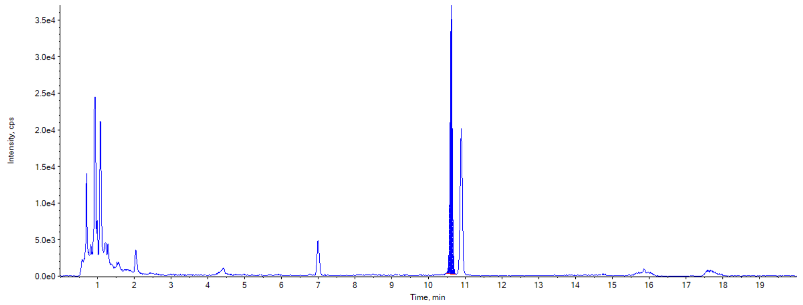

462.200/282.300 Da

ZOOMED

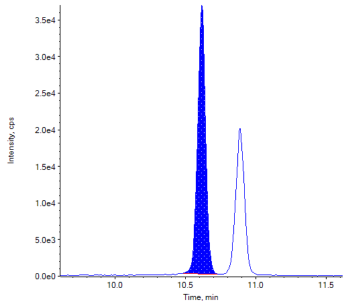

IS_GALSPH(D18:1) (Area 2.81e+005 cps; RT 10.9 min; 467.300/287.300 Da)

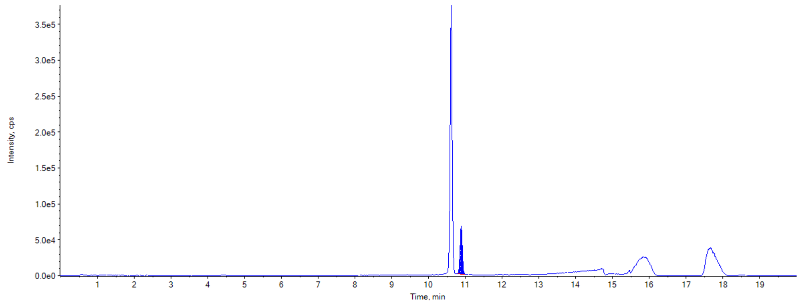

467.300/287.300 Da

ZOOMED

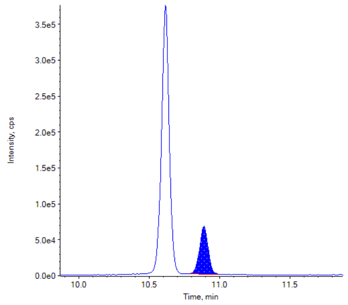

IS_GLUSPH(D18:1) (Area 1.42e+006 cps; RT 10.6 min; 467.300/287.300 Da)

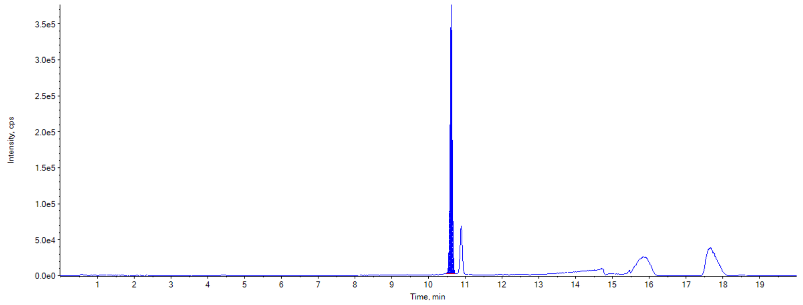

467.300/287.300 Da

ZOOMED

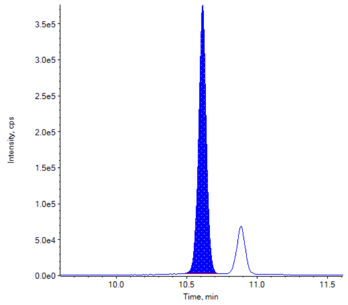

Sample Name	Q14329-011_QC-L	Sample Type	Quality Control
Sample ID	1700747-847126	Dilution	1.00
File Name	21287-AB011\21287-AB011.wiff	Acquisition date	2021-12-22 23:50:48

GALSPH(D18:1) (0.0306 pmol/mL; Area 1.61e+004 cps; RT 10.8 min)

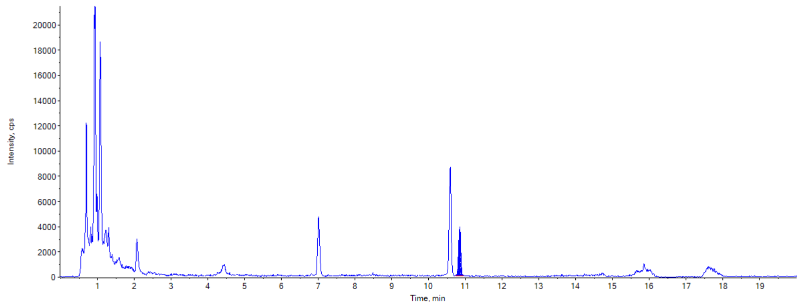

462.200/282.300 Da

ZOOMED

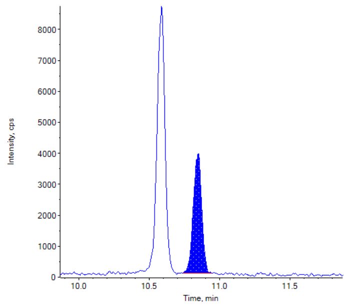

GLUSPH(D18:1) (0.0313 pmol/mL; Area 3.38e+004 cps; RT 10.6 min)

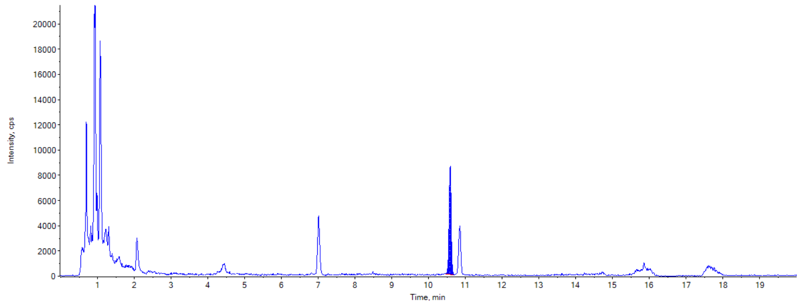

462.200/282.300 Da

ZOOMED

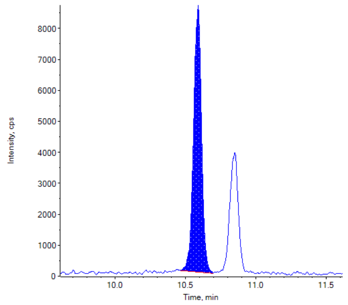

IS_GALSPH(D18:1) (Area 2.64e+005 cps; RT 10.8 min; 467.300/287.300 Da)

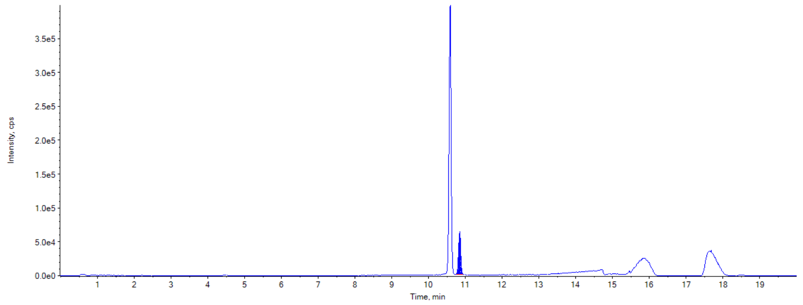

467.300/287.300 Da

ZOOMED

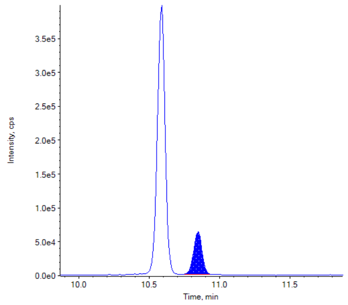

IS_GLUSPH(D18:1) (Area 1.52e+006 cps; RT 10.6 min; 467.300/287.300 Da)

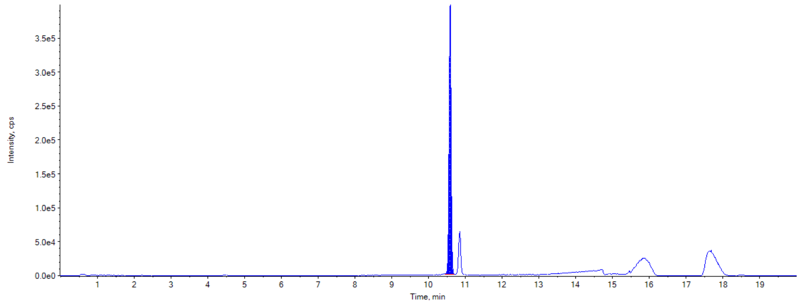

467.300/287.300 Da

ZOOMED

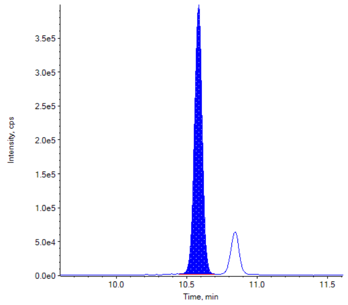

Sample Name	Q14440-003_QC-P	Sample Type	Pooled Quality Control
Sample ID	1700231-847130	Dilution	1.00
File Name	21287-AB011\21287-AB011.wiff	Acquisition date	2021-12-23 00:21:23

GALSPH(D18:1) (0.183 pmol/mL; Area 7.04e+004 cps; RT 10.9 min)

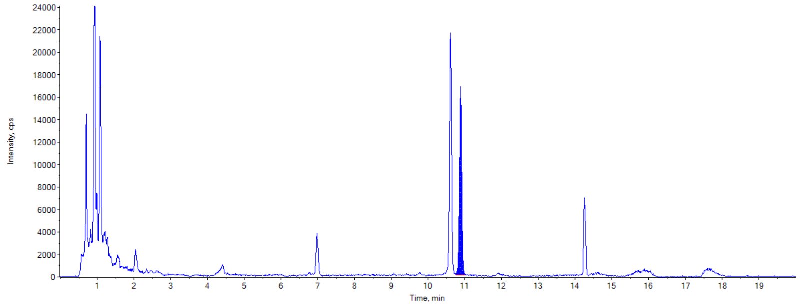

462.200/282.300 Da

ZOOMED

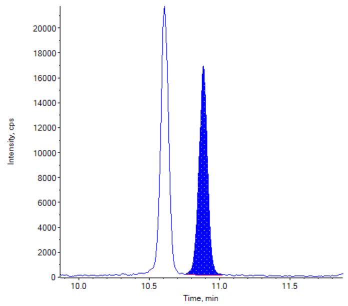

GLUSPH(D18:1) (0.114 pmol/mL; Area 8.35e+004 cps; RT 10.6 min)

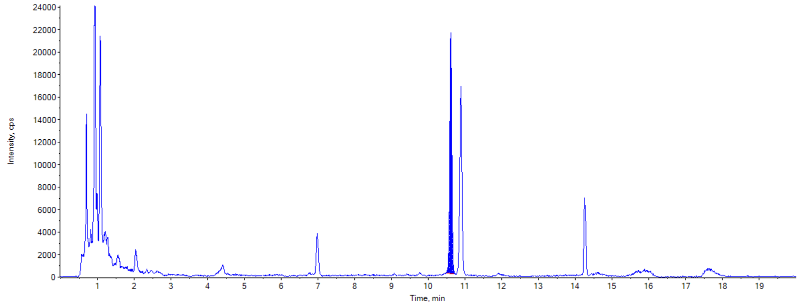

462.200/282.300 Da

ZOOMED

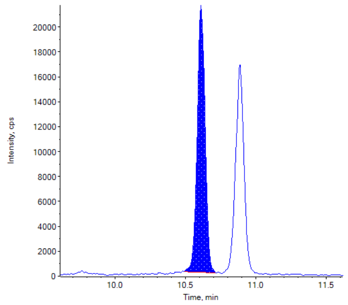

IS_GALSPH(D18:1) (Area 1.93e+005 cps; RT 10.9 min; 467.300/287.300 Da)

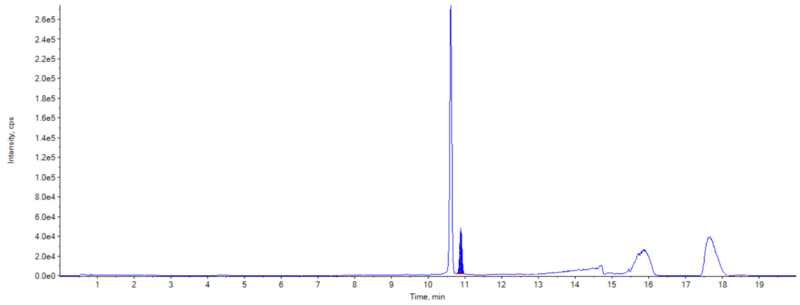

467.300/287.300 Da

ZOOMED

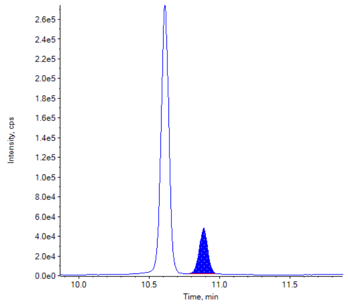

IS_GLUSPH(D18:1) (Area 1.05e+006 cps; RT 10.6 min; 467.300/287.300 Da)

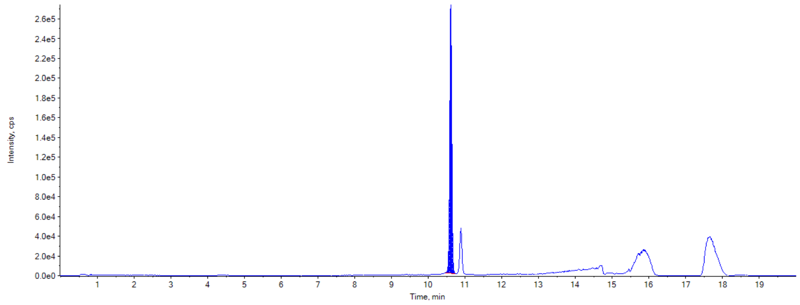

467.300/287.300 Da

ZOOMED

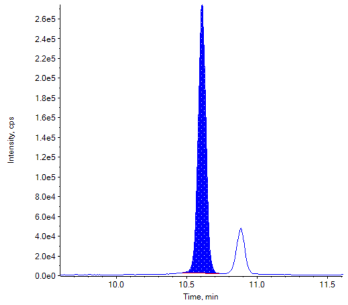

Low GluSph Samples N=2

	Sample ID	GluSph (pmol/ml)	GalSph (pmol/ml)
1	1675301-846581	0.00898	0.148
2	1675305-846585	0.00517	0.103

Sample Name	ID31557-FR20547458	Sample Type	Unknown
Sample ID	1675301-846581	Dilution	1.33
File Name	21287-AB011\21287-AB011.wiff	Acquisition date	2021-12-23 01:53:04

GALSPH(D18:1) (0.148 pmol/mL; Area 4.94e+004 cps; RT 10.9 min)

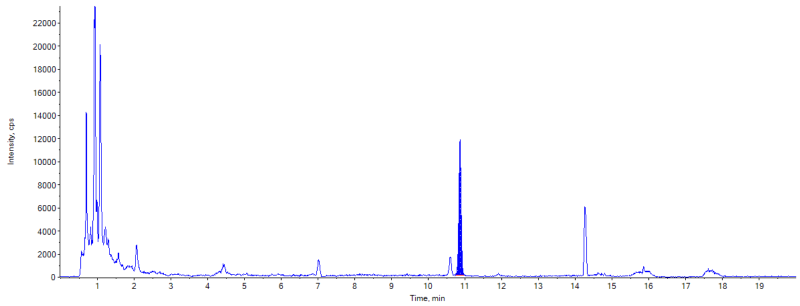

462.200/282.300 Da

ZOOMED

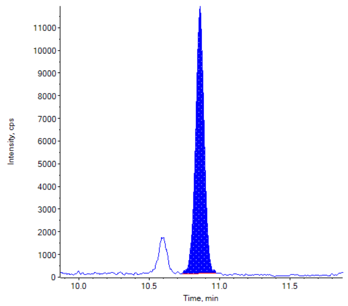

GLUSPH(D18:1) (0.00898 pmol/mL; Area 6.31e+003 cps; RT 10.6 min)

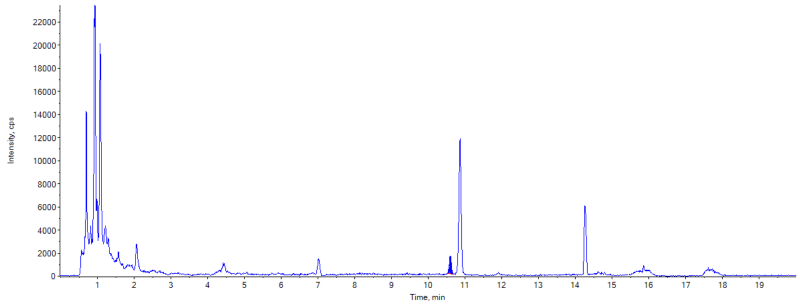

462.200/282.300 Da

ZOOMED

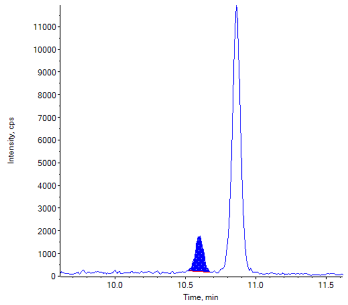

IS_GALSPH(D18:1) (Area 2.22e+005 cps; RT 10.9 min; 467.300/287.300 Da)

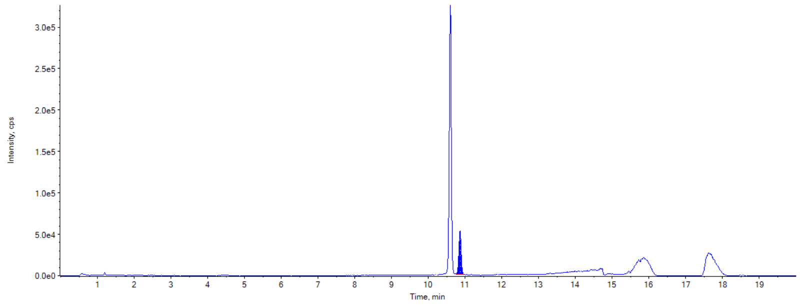

467.300/287.300 Da

ZOOMED

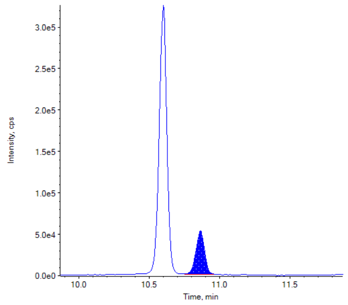

IS_GLUSPH(D18:1) (Area 1.23e+006 cps; RT 10.6 min; 467.300/287.300 Da)

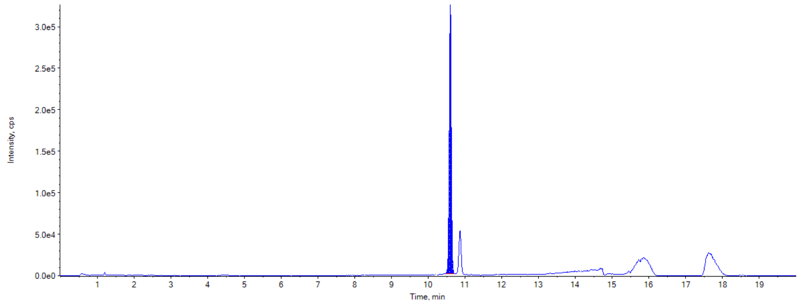

467.300/287.300 Da

ZOOMED

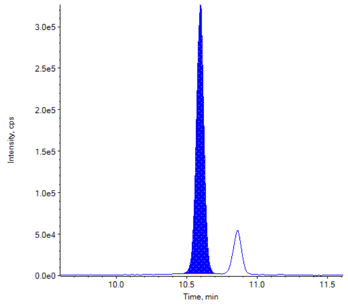

Sample Name	ID27111-FR21346180	Sample Type	Unknown
Sample ID	1675305-846585	Dilution	1.00
File Name	21287-AB011\21287-AB011.wiff	Acquisition date	2021-12-23 03:55:18

GALSPH(D18:1) (0.103 pmol/mL; Area 4.05e+004 cps; RT 10.9 min)

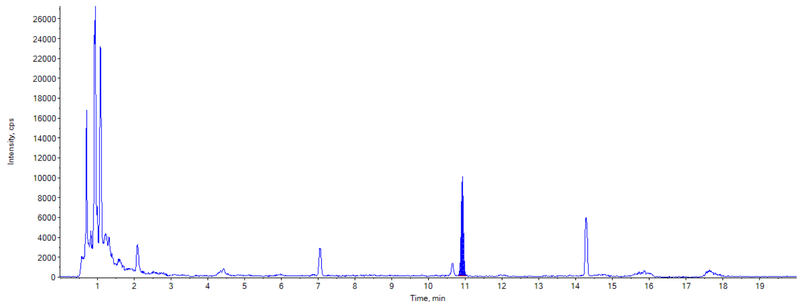

462.200/282.300 Da

ZOOMED

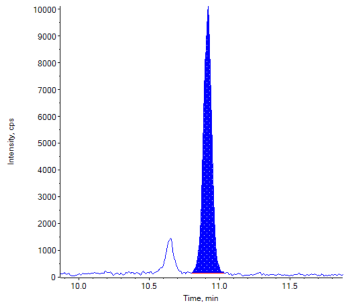

GLUSPH(D18:1) (0.00517 pmol/mL; Area 4.67e+003 cps; RT 10.7 min)

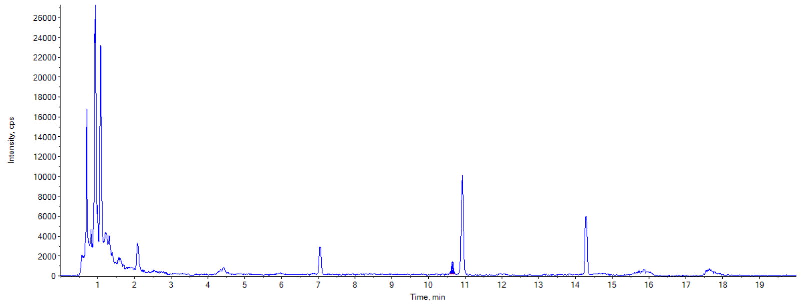

462.200/282.300 Da

ZOOMED

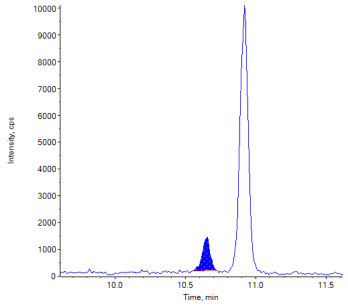

IS_GALSPH(D18:1) (Area 1.96e+005 cps; RT 10.9 min; 467.300/287.300 Da)

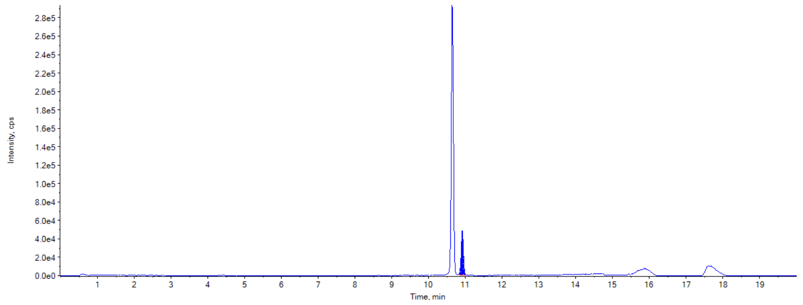

467.300/287.300 Da

ZOOMED

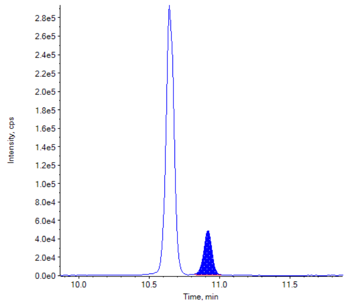

IS_GLUSPH(D18:1) (Area 1.15e+006 cps; RT 10.6 min; 467.300/287.300 Da)

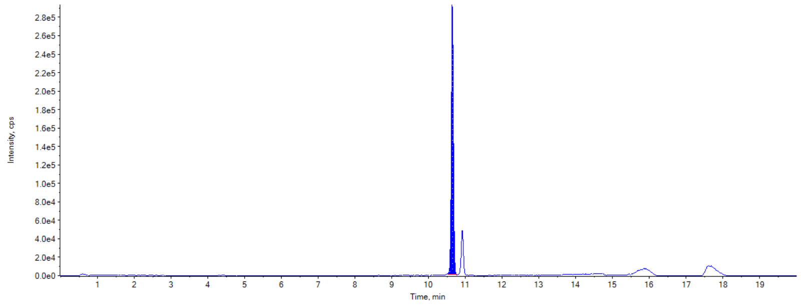

467.300/287.300 Da

ZOOMED

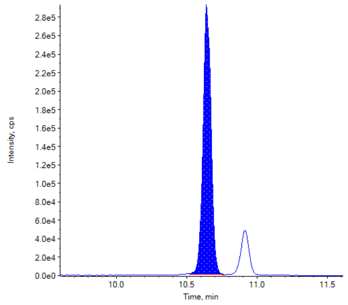

High GluSph Samples N=2

	Sample ID	GluSph (pmol/ml)	GalSph (pmol/ml)
1	1700760-847127	0.110	0.157
2	1700761-847128	0.114	0.155

Sample Name	Q14440-003_QC-P	Sample Type	Quality Control
Sample ID	1700760-847127	Dilution	1.00
File Name	21287-AB011\21287-AB011.wiff	Acquisition date	2021-12-23 07:59:48

GALSPH(D18:1) (0.157 pmol/mL; Area 6.92e+004 cps; RT 10.9 min)

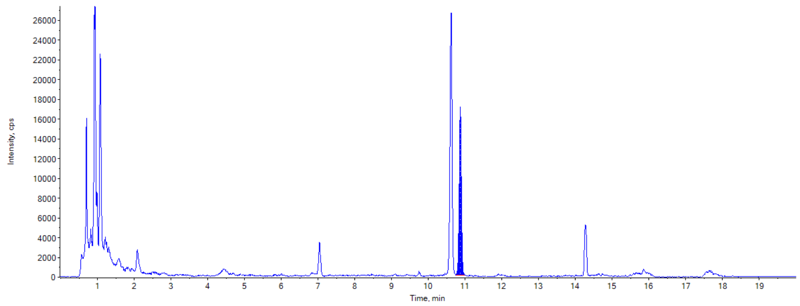

462.200/282.300 Da

ZOOMED

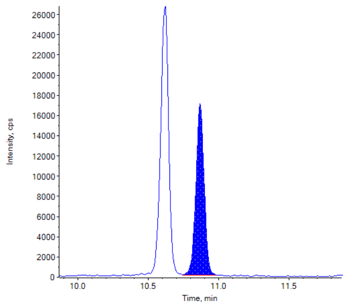

GLUSPH(D18:1) (0.110 pmol/mL; Area 1.04e+005 cps; RT 10.6 min)

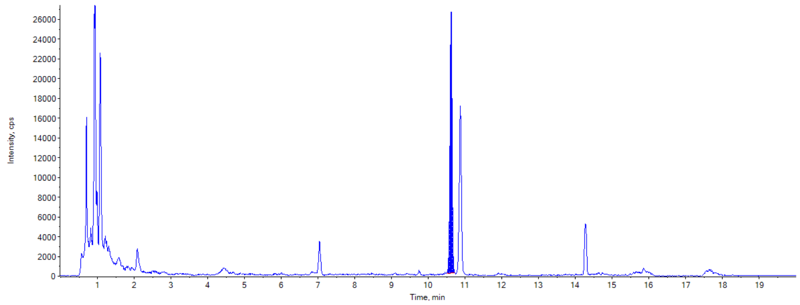

462.200/282.300 Da

ZOOMED

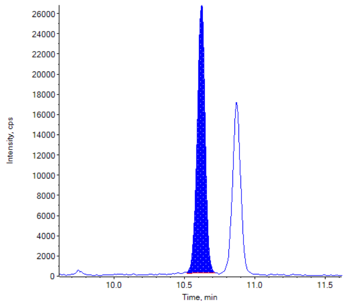

IS_GALSPH(D18:1) (Area 2.21e+005 cps; RT 10.9 min; 467.300/287.300 Da)

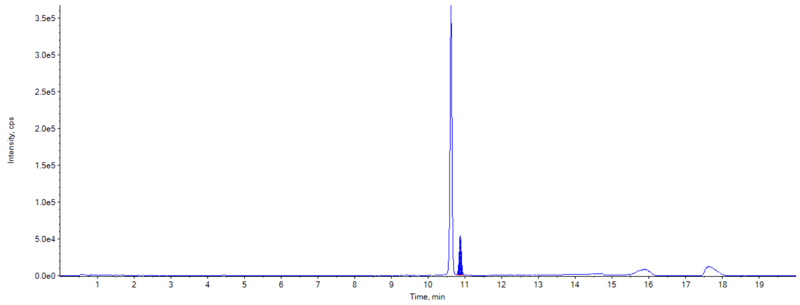

467.300/287.300 Da

ZOOMED

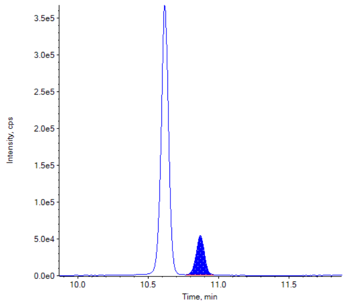

IS_GLUSPH(D18:1) (Area 1.35e+006 cps; RT 10.6 min; 467.300/287.300 Da)

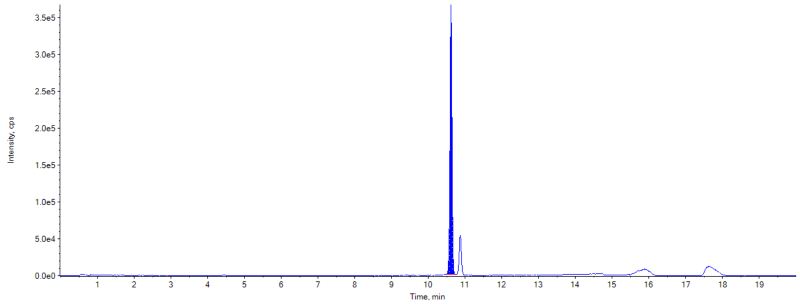

467.300/287.300 Da

ZOOMED

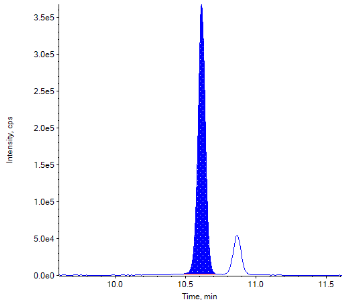

Sample Name	Q14440-003_QC-P	Sample Type	Quality Control
Sample ID	1700761-847128	Dilution	1.00
File Name	21287-AB011\21287-AB011.wiff	Acquisition date	2021-12-23 15:07:42

GALSPH(D18:1) (0.155 pmol/mL; Area 6.41e+004 cps; RT 10.9 min)

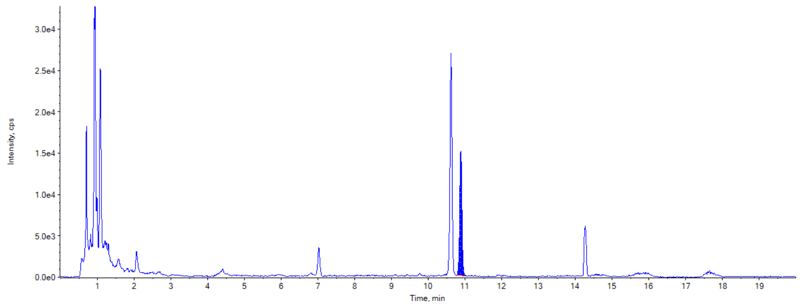

462.200/282.300 Da

ZOOMED

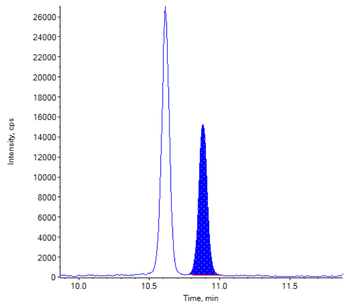

GLUSPH(D18:1) (0.114 pmol/mL; Area 1.03e+005 cps; RT 10.6 min)

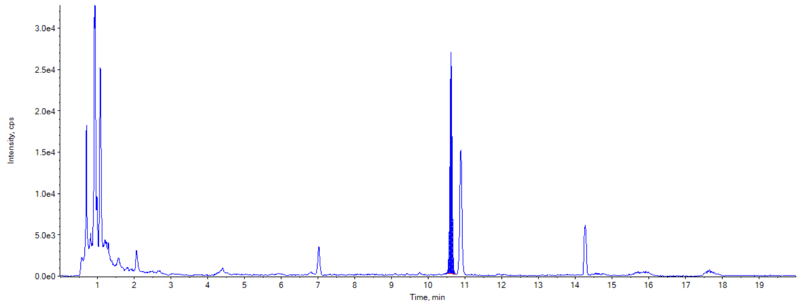

462.200/282.300 Da

ZOOMED

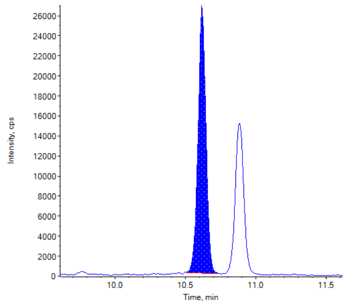

IS_GALSPH(D18:1) (Area 2.07e+005 cps; RT 10.9 min; 467.300/287.300 Da)

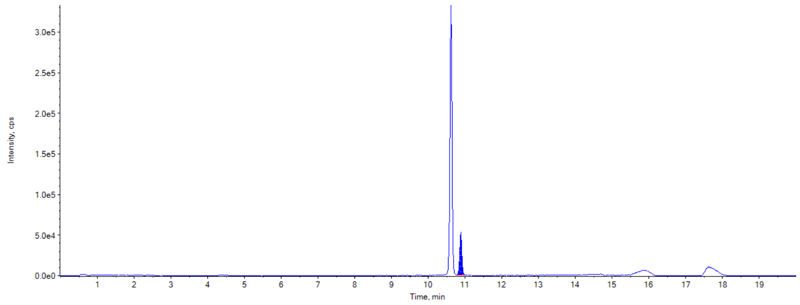

467.300/287.300 Da

ZOOMED

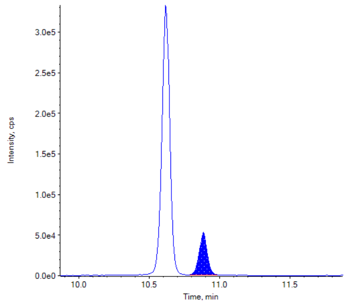

IS_GLUSPH(D18:1) (Area 1.30e+006 cps; RT 10.6 min; 467.300/287.300 Da)

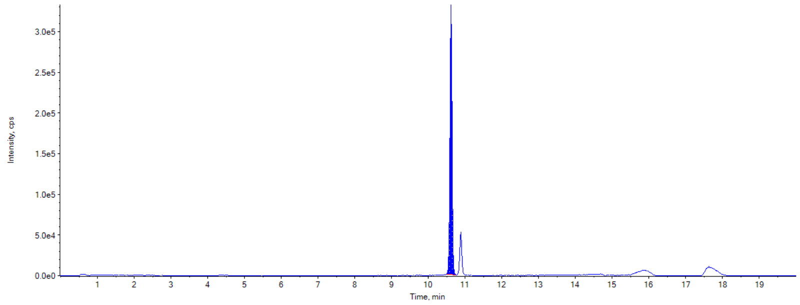

467.300/287.300 Da

ZOOMED

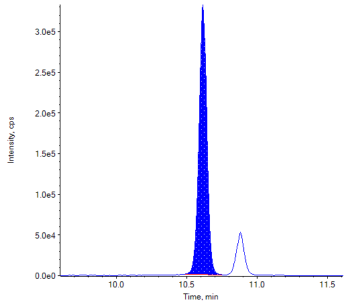

CONSORT 2010 checklist of information to include when reporting a randomised trial*

Section/Topic	Item No	Checklist item	Reported on page No
**Title and abstract**			
	1a	Identification as a randomised trial in the title	Not applicable
	1b	Structured summary of trial design, methods, results, and conclusions (for specific guidance see CONSORT for abstracts)	Not applicable
**Introduction**			
Background and objectives	2a	Scientific background and explanation of rationale	3
	2b	Specific objectives or hypotheses	3+4
**Methods**			
Trial design	3a	Description of trial design (such as parallel, factorial) including allocation ratio	Not applicable
	3b	Important changes to methods after trial commencement (such as eligibility criteria), with reasons	Not applicable
Participants	4a	Eligibility criteria for participants	7
	4b	Settings and locations where the data were collected	7
Interventions	5	The interventions for each group with sufficient details to allow replication, including how and when they were actually administered	Not applicable
Outcomes	6a	Completely defined pre-specified primary and secondary outcome measures, including how and when they were assessed	7-11
	6b	Any changes to trial outcomes after the trial commenced, with reasons	Not applicable
Sample size	7a	How sample size was determined	Not applicable
	7b	When applicable, explanation of any interim analyses and stopping guidelines	
Randomisation:			
Sequence generation	8a	Method used to generate the random allocation sequence	Not applicable
	8b	Type of randomisation; details of any restriction (such as blocking and block size)	Not applicable
Allocation concealment mechanism	9	Mechanism used to implement the random allocation sequence (such as sequentially numbered containers), describing any steps taken to conceal the sequence until interventions were assigned	Not applicable
Implementation	10	Who generated the random allocation sequence, who enrolled participants, and who assigned participants to interventions	Not applicable
Blinding	11a	If done, who was blinded after assignment to interventions (for example, participants, care providers, those	Not applicable

		assessing outcomes) and how	
	11b	If relevant, description of the similarity of interventions	
Statistical methods	12a	Statistical methods used to compare groups for primary and secondary outcomes	11
	12b	Methods for additional analyses, such as subgroup analyses and adjusted analyses	11
**Results**			
Participant flow (a diagram is strongly recommended)	13a	For each group, the numbers of participants who were randomly assigned, received intended treatment, and were analysed for the primary outcome	7
	13b	For each group, losses and exclusions after randomisation, together with reasons	Not applicable
Recruitment	14a	Dates defining the periods of recruitment and follow-up	Not apllicable
	14b	Why the trial ended or was stopped	Not applicable
Baseline data	15	A table showing baseline demographic and clinical characteristics for each group	Supplementary material
Numbers analysed	16	For each group, number of participants (denominator) included in each analysis and whether the analysis was by original assigned groups	Not applicable
Outcomes and estimation	17a	For each primary and secondary outcome, results for each group, and the estimated effect size and its precision (such as 95% confidence interval)	4+5
	17b	For binary outcomes, presentation of both absolute and relative effect sizes is recommended	Not applicable
Ancillary analyses	18	Results of any other analyses performed, including subgroup analyses and adjusted analyses, distinguishing pre-specified from exploratory	Not applicable
Harms	19	All important harms or unintended effects in each group (for specific guidance see CONSORT for harms)	Not applicable
**Discussion**			
Limitations	20	Trial limitations, addressing sources of potential bias, imprecision, and, if relevant, multiplicity of analyses	6+7
Generalisability	21	Generalisability (external validity, applicability) of the trial findings	6
Interpretation	22	Interpretation consistent with results, balancing benefits and harms, and considering other relevant evidence	5
**Other information**			
Registration	23	Registration number and name of trial registry	Not applicable
Protocol	24	Where the full trial protocol can be accessed, if available	Not applicable
Funding	25	Sources of funding and other support (such as supply of drugs), role of funders	12

Citation: Schulz KF, Altman DG, Moher D, for the CONSORT Group. CONSORT 2010 Statement: updated guidelines for reporting parallel group randomised trials. BMC Medicine. 2010;8:18.
© 2010 Schulz et al. This is an Open Access article distributed under the terms of the Creative Commons Attribution License (http://creativecommons.org/licenses/by/2.0), which permits unrestricted use, distribution, and reproduction in any medium, provided the original work is properly cited.

*We strongly recommend reading this statement in conjunction with the CONSORT 2010 Explanation and Elaboration for important clarifications on all the items. If relevant, we also recommend reading CONSORT extensions for cluster randomised trials, non-inferiority and equivalence trials, non-pharmacological treatments, herbal interventions, and pragmatic trials. Additional extensions are forthcoming: for those and for up-to-date references relevant to this checklist, see www.consort-statement.org.
